# Molecular signatures associated with tumor-specific immune response in melanoma patients treated with dendritic cell-based immunotherapy

**DOI:** 10.18632/oncotarget.24795

**Published:** 2018-03-30

**Authors:** Tamara García-Salum, Andrea Villablanca, Franziska Matthäus, Andrés Tittarelli, Mauricio Baeza, Cristián Pereda, M. Alejandra Gleisner, Fermín E. González, Mercedes N. López, Jörg D. Hoheisel, Johannes Norgauer, Peter J. Gebicke-Haerter, Flavio Salazar-Onfray

**Affiliations:** ^1^ Disciplinary Program of Immunology, Institute of Biomedical Sciences, Faculty of Medicine, Universidad de Chile, 8380453 Santiago, Chile; ^2^ Millennium Institute on Immunology and Immunotherapy, Faculty of Medicine, Universidad de Chile, 8380453 Santiago, Chile; ^3^ Faculty of Biological Sciences and FIAS, University of Frankfurt, Ruth-Moufang-Straße 1, 60438 Frankfurt am Main, Germany; ^4^ Laboratory of Periodontal Biology, Faculty of Dentistry, Universidad de Chile, 8380492 Santiago, Chile; ^5^ Laboratory of Experimental Immunology and Cancer, Faculty of Dentistry, Universidad de Chile, 8380492 Santiago, Chile; ^6^ Functional Genome Analysis, German Cancer Research Centre (DKFZ), Im Neuenheimer Feld 580, 69120 Heidelberg, Germany; ^7^ Department of Dermatology, Jena University Hospital D-07743 Jena, Germany; ^8^ Institute of Psychopharmacology, Central Institute of Mental Health, University of Heidelberg, J5, 68159 Mannheim, Germany

**Keywords:** molecular signatures, immunotherapy, melanoma, CXCR4, CD32

## Abstract

**Purpose:**

We previously showed that autologous dendritic cells (DCs) loaded with an allogeneic heat shock (HS)-conditioned melanoma cell-derived lysate, called TRIMEL, induce T-cell-mediated immune responses in stage IV melanoma patients. Importantly, a positive delayed-type hypersensitivity (DTH) reaction against TRIMEL after vaccination, correlated with patients prolonged survival. Furthermore, we observed that DTH reaction was associated with a differential response pattern reflected in the presence of distinct cell subpopulations in peripheral blood. Detected variations in patient responses encouraged molecular studies aimed to identify gene expression profiles induced after vaccination in treated patients, allowing the identification of new molecular predictive markers.

**Methods:**

Gene expression patterns were analyzed by microarrays during vaccination, and some of them confirmed by quantitative real-time reverse transcriptase PCR (qRT-PCR) in the total leukocyte population of a representative group of responder and non-responder patients. New candidates for biomarkers with predictive value were identified using bioinformatics, molecular analysis, and flow cytometry.

**Results:**

Seventeen genes overexpressed in responder patients after vaccination respect to non-responders were identified after a mathematical analysis, from which ten were linked to immune responses and five related to cell cycle control and signal transduction. In immunological responder patients, increased protein levels of the chemokine receptor CXCR4 and the Fc-receptor CD32 were observed on cell membranes of CD8+ T and B cells and the monocyte population, respectively, confirming gene expression results.

**Conclusions:**

Our study contributes to finding new molecular markers associated with clinical outcome and better understanding of clinically relevant immunological responses induced by anti-tumor DC-vaccines.

## INTRODUCTION

Influence of genetic predisposition in medical conditions is a relevant aspect to be evaluated related to the benefit of a specific drug or therapeutic approach. A patient's genetic predisposition or resistance can be revealed, at least in part, by high-throughput gene expression profile studies, including microarrays, in which gene expression can be determined, preferentially in an extended manner [1–3].

Recently, immunotherapy has emerged as a reliable therapeutic alternative after decades of treating cancer mostly by chemo- and radiotherapy [4]. The accumulated knowledge regarding immunological mechanisms involved in cancer dynamics has paved the way for novel therapeutic approaches that manipulate a patient's immune system to control the disease [5]. These approaches include the use of: proinflammatory cytokines; fully humanized monoclonal antibodies (mAbs) directed against check-point molecules such as anti-CTLA4 and anti-PD-1/PD-L1 [6]; cell-based immunotherapies, such as adoptive transfer of CD8+ T lymphocytes [7, 8]; and several cancer vaccines, including autologous dendritic cell (DC)-based vaccines [9, 10]. Regarding immunization with DCs, several different protocols including the use of DCs, transfected with tumor associated antigen (TAA)- and immunomodulatory molecules-derived mRNA [11–13], or loaded with tumor cell lysates [9, 10, 14], among others, have been extensively explored. Despite the abilities of DC-vaccines' for stimulating the antitumor response and trigger the generation of immunological memory, in some cancer, an important percentage of patients treated with DC-based immunotherapies remain refractory to these approaches. In fact, individual factors involved in the differential capacity of patients to respond to immunizations remain poorly understood.

For the last ten years, a protocol using a unique DC-based vaccine, consisting in autologous monocyte-derived DCs loaded with an allogeneic heat shock (HS)-conditioned melanoma cell-derived lysate (called TRIMEL), obtained from three allogeneic melanoma cell lines, has been included in phase I/II clinical trials. In this approach TRIMEL lysate provides a reproducible pool of several potential TAAs to DCs, suitable to be used in a broad number of patients, independently of their major histocompatibility complex (MHC) haplotype or the availability of autologous tumor tissue. Moreover, the unique composition of TRIMEL after heat conditioning allows for danger signals to trigger the optimal maturation and activation of monocyte-derived DCs [10,14]. Moreover, lysate-loaded DCs can induce specific *in vitro* and *in vivo* antitumor and memory immune responses in patients with advanced malignant melanoma. Our clinical data showed that more than 60% of DC-vaccinated patients develop a delayed-type hypersensitivity (DTH) response against antigens derived from TRIMEL, and were classified as immunological responder patients, showing a significant correlation with improved survival of stage IV melanoma patients (median: 33 months for DTH-positive) compared with a median of 11 months for stage IV/DTH-negative patients that were classified as non-responder patients [9, 14]. In those cases, DTH corresponds to an inflammatory tissue reaction that reflects a cell-mediated immune response against tumor associated antigens. Importantly, immunological responder patients develop an inflammatory response during the treatment, observed by increased Th1 and Th17 effector lymphocyte subsets in peripheral blood, while non-responder patients have a significant increase in Th3 regulatory T cells [15]. Moreover, the presence of a specific single nucleotide polymorphism (896 A>G) in the TLR4 gene reduces the ability of TRIMEL-loaded DCs to activate anti-tumor specific T cells. This data is also associated with a diminished median survival of the treated patients bearing the mutant allele of TLR4 [16]. Taken together, these findings support the existence of differences in gene and/or protein expression profiles of treated melanoma patients induced by TRIMEL-loaded DCs-based immunotherapy, either before, during and/or after repeated immunizations. Specific gene expression profiles obtained of responder and non-responder patients could help identifying gene signature modules that may act as predictive biomarkers for an effective anti-melanoma immune response [17].

In this work, we quantified gene and protein expression of advanced melanoma patients in response to the treatment with TRIMEL-loaded DCs, according to the timing of their treatment. Our results showed that seventeen genes were consistently overexpressed in responder patients during the vaccination protocol, and the protein products of ten of those genes have well-known immune-response-related functions. Importantly, we observed that the chemokine receptor CXCR4 and the receptor for the Fc portion of IgG, CD32, were overexpressed in the lymphocytes' cell membranes and in the monocyte population in immunological responder patients. Further studies are warranted to confirm these targets as predictive and/or follow-up biomarkers for melanoma patients.

## RESULTS

### Clinical characteristics of TRIMEL-loaded DCs-vaccinated patients

**Figure 1:**
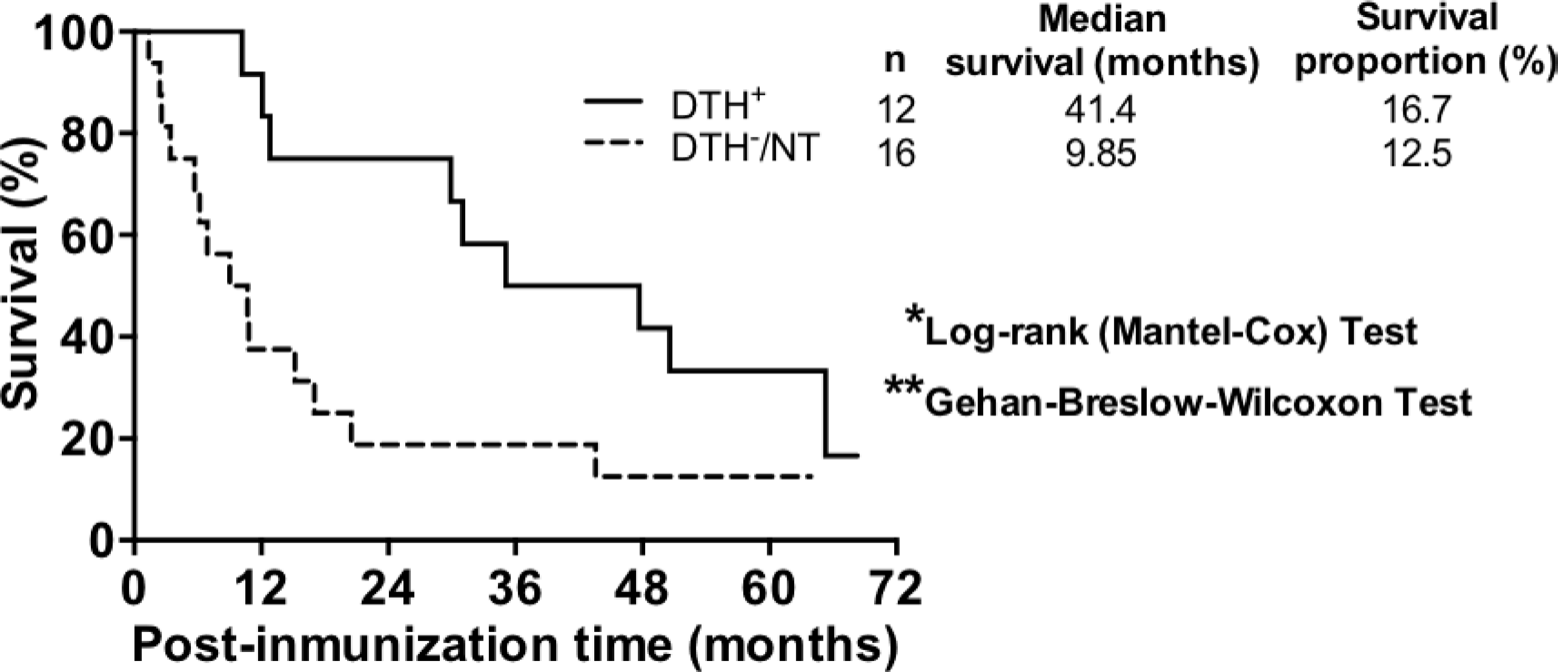
TRIMEL-loaded DCs-treated immunological responder patients have a higher median survival than non-immunological responder patients Post-TRIMEL-loaded DCs immunotherapy overall survival curves for immunological responder (DTH+: full line) and immunological non-responder (DTH-/NT: dash line) patients. ^*^p < 0.05 ^**^p < 0.01. NT: Not tested.

Twenty-eight patients with advanced malignant melanoma (7 stage III and 21 stage IV patients) were recruited and treated with TRIMEL-loaded DCs according to a previously described protocol [9]. Peripheral blood samples were collected from patients at each time point during the treatment, i.e., at leukapheresis, at each inoculation step (1st to 4th vaccination) and at the evaluation of DTH response to TRIMEL lysate, one month after the end of the vaccine protocol. Twelve patients displayed a positive cellular immune response to the therapy, which means a DTH reaction resulting in a skin erythema or induration with a diameter greater than or equal to 5 mm against TRIMEL-derived melanoma antigens (DTH+). This group of patients was considered as immunological responders. On other hand, eleven patients did not reacted or displayed a DTH less than 5 mm diameter after vaccination (DTH-) and five died before vaccination protocol ending without showing any signal of induration at vaccination site during the protocol, which were consequently considered as immunologically non-responders to therapy (16 patients in total) (Table 1). At the time of completion of the study (December 2013), 3 out of 12 immunological responder patients were alive (survival rate: 16.7%; median survival time: 41.4 months), and 2 out of the 16 non-immunological responders were alive (survival rate: 12.5%; median survival time: 9.85 months; Figure 1). These results are consistent with previous data obtained from over 100 stage IV melanoma patients and are in line with the immune cell response generated by TRIMEL-loaded DCs immunotherapy (post-therapy median overall survival time: 33 vs. 11 months for DTH+ and DTH– patients, respectively) [9, 14].

**Table 1:** Clinical characteristics of the TRIMEL-loaded DCs treated melanoma patients

Patient code	Gender	AJCC stage	Primary tumor localization	Metastasis	Additional treatment	DTH response	Overall survival (months)
MT061^p^	M	IV	Urethral meatus	M1c	CTX	–	10.8^†^
MT065^p^	F	IV	Scalp	M1c	CTX	–	5.7^†^
MT066^p^	M	IIIC	Abdominal wall	ND	DTIC, RT	+	47.7^†^
MT072^p^	M	IV	Dorsal	M1c	NA	–	15.2^†^
MT076^p^	F	IV	Left foot	M1b	RT	+	35.1^†^
MT079^p,g^	F	IV	Left flank	M1c	Surgery	–	9^†^
MT080^p,g^	M	IV	Palate	M1c	Surgery, RT	+	68.3
MT083^g^	M	IIIC	Right hand	N4	Surgery	–	17^†^
MT084^g^	M	IIIC	Right foot	N4	Surgery	+	65.3^†^
MT087^g^	M	IV	Right hand	M1b	Surgery, RT	NT	1.4^†^
MT089^g^	M	IV	Right foot	M1b	Surgery, RT	NT	6.2^†^
MT091^p,g^	M	IV	Scalp	M1c	Surgery, RT	+	50.6^†^
MT093^g^	F	IV	Uveal melanoma	M1c	Surgery	+	64.9
MT094^g^	M	IV	Head/Neck	M1c	Surgery	NT	3.4^†^
MT096^g^	M	IIIA	Dorsal	N2a	Surgery	+	31^†^
MT098^g^	M	IV	Right hand	M1c	Surgery	NT	2.6^†^
MT101^g^	M	IV	Dorsal/Right leg	M1a	Surgery	–	63.9
MT105^p^	F	IIIA	Right leg	N2a	NA	–	63.5
MT109^p^	F	IV	ND	M1c	Surgery	NT	2.4^†^
MT114^p^	M	IV	Thoracic wall	M1c	RT, IFN-α	–	10.7^†^
MT122^p^	M	IIIC	Left leg	ND	NA	+	29.9^†^
MT123^p^	M	IV	Left flank	M1c	RT	–	6.9^†^
MT127^p^	F	IV	Left foot	M1b	NA	+	12.1^†^
MT128^p^	F	IIIB	Right shoulder	N2c	RT	+	56.5
MT132^p^	M	IV	Uveal melanoma	M1c	DTIC	–	43.6^†^
MT141^p^	M	IV	Knee	M1c	RT	–	20.5^†^
MT147^p^	F	IV	Left leg	M1b	RT	+	12.8^†^
MT158^p^	M	IV	Right shoulder	M1a	Surgery, RT	+	10.2^†^

Abbreviations: p, protein expression analysis; g, gen expression analysis; F, Female; M, Male; AJCC, American Joint Committee on Cancer; ND, Not determined; RT, Radiotherapy; CTX, Chemotherapy; DTIC, Dacarbazine; NA, Not apply; NT, Not tested; ^†^, Deceased.

### Gene-expression temporal behavior of TRIMEL-loaded DCs-treated patients

Transcriptome profiling of twelve melanoma patients (from MT079 to MT101, Table 1) was assessed by microarray analysis. In total 72 samples were expected to be collected (6 per patient) during the immunization protocol. However, only 46 samples out 72 were obtained due to some patients did not survive the complete treatment or their clinical condition did not permit that samples from all time points could be collected (Supplementary Table 1).

To determine whether there were differences at gene expression levels in patient's peripheral blood during the immunotherapy, we reclassified our patients according to their immunological response and survival times. One group of clinical responder patients, 7 in total, comprised all DTH+ patients and also two DTH- patients (MT083 and MT101) that showed considerably prolonged survival times (17 and 63.9 months, respectively), which even exceed the reported median survival obtained by stage IV/DTH-negative patients in a previously reported study (11 months; SD 4.24 and 7.78, respectively) [9]. A second group, non-responder patients, included a total of 5 patients, considered only immunological non- responder patients to therapy, all of them with shorter survival times than 11 months. For the analysis, mathematical rules were defined to compare between patients relative gene expression during the vaccination processes; at the beginning (before 1st and 2nd vaccination), during (before 3rd vaccination), and at the end (before 4th vaccination and DTH assessment). Applied mathematical rules are in form of a set of scores for each gene as explained in Materials and Methods section, (Time-course analysis).

**Figure 2:**
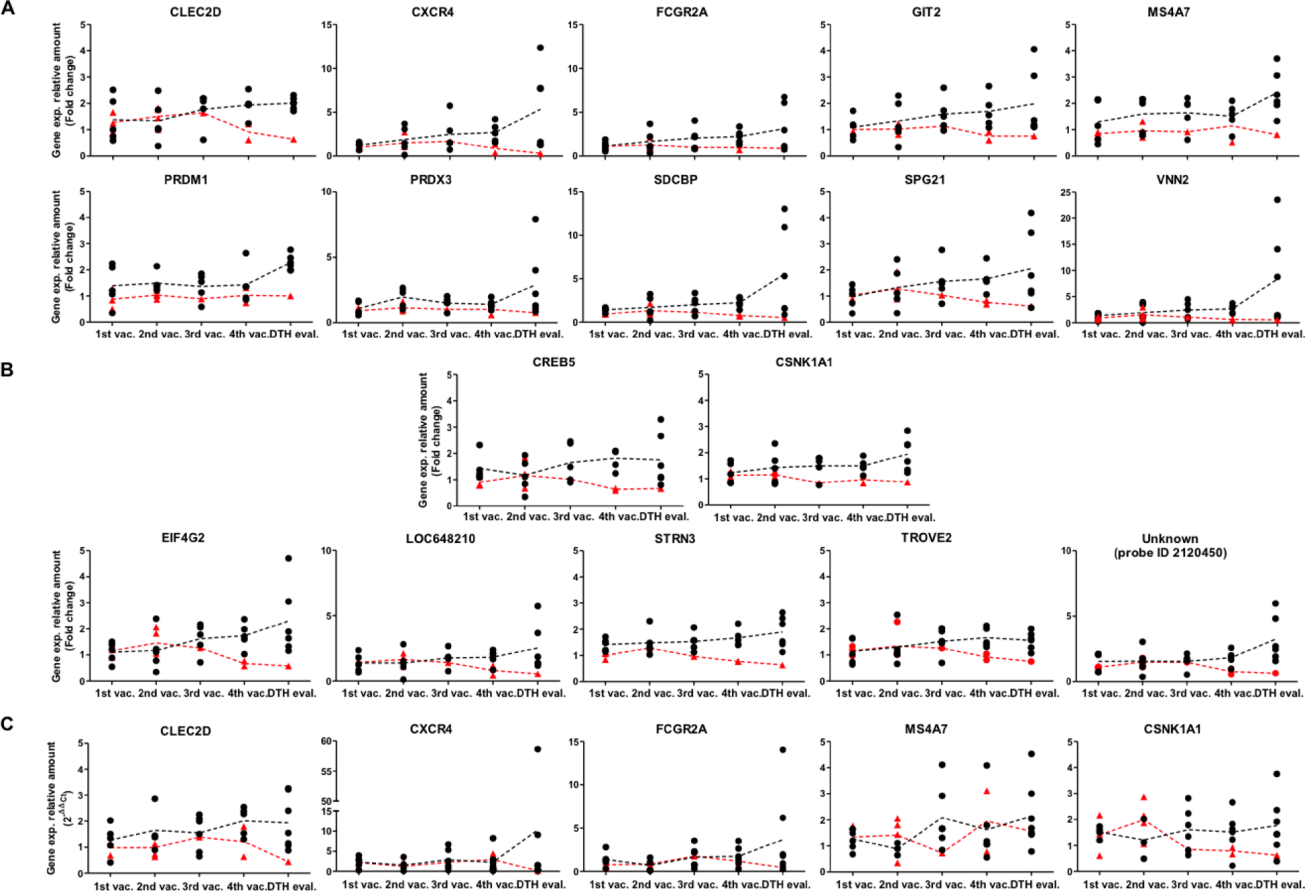
Differences in temporal gene expression of potential molecular markers between clinical responder and non-responder patients Vaccinated patients were reclassified according DTH reaction and overall survival as described in Material and methods. (**A**) Relative gene expression of genes *CLEC2D, CXCR4, FCGR2A, GIT2, MS4A7, PRDM1, PRDX3, SDCBP, SPG21* and *VNN2* were determined by microarrays. Black dots: Clinical responders (DTH^+^/long survivor patients); samples from 1st-4th vaccination (*n* = 5), samples from DTH evaluation (*n* = 6); red triangles: Non-responder patients (DTH^−^ patients), samples from 1st vaccination (*n* = 3), 2nd vaccination (*n* = 4), 3rd vaccination (*n* = 1), 4th vaccination (*n* = 2) and DTH evaluation (*n* = 1). (**B**) Relative expression of genes *CREB5, CSNK1A1, EIF4G2, LOC648210, STRN3, TROVE2* and unknown (probe ID 2120450) were determined by microarrays. Black dots: Clinical responder patients, samples from 1st to 4th vaccination (*n* = 5), DTH evaluation (*n* = 6); red triangles: Non-responder patients, samples from 1st vaccination (*n* = 3), 2nd vaccination (*n* = 4), 3rd vaccination (*n* = 1). 4th vaccination (*n* = 2) and DTH evaluation (*n* = 1). (**C**) Relative gene expression determined by qRT-PCR of genes *CLEC2D, CXCR4, FCGR2A, MS4A7* and *CSNK1A1*. Black dots: Clinical responder patients, samples from 1st vaccination (*n* = 4–6), 2nd vaccination (*n* = 3–4), 3rd vaccination (*n* = 6), 4th vaccination (*n* = 5–7), and DTH evaluation (*n* = 7); red triangles: non-responder patients, samples from 1st vaccination (*n* = 2–3), 2nd vaccination (*n* = 4); 3rd vaccination (*n* = 1), 4th vaccination (*n* = 2) and DTH evaluation (*n* = 1). Fold changes relative to the respective reference sample are given. Each data dot represents one patient's sample, each line represents the mean of the data dots. Vaccination (vac.); evaluation (eval.); ID, identification number.

In this way, time courses of 17 genes were identified that allowed us to distinguish between both groups of patients. Fifteen of these genes have known functions. Interestingly, ten (66.7%) are related to the immune response—CLEC2D, CXCR4, FCGR2A, GIT2, MS4A7, PRDM1, PRDX3, SDCBP, SPG21 and VNN2— whereas five (33.3%) are associated with cell cycle and signal transduction—CREB5, CSNK1A1, EIF4G2, STRN3 and TROVE2 (Figure 2A, 2B and Supplementary Table 2). Some of the genes associated with the immune response, showed expression differences beginning at early time points (before 3rd vaccination), such as MS4A7, PRDM1 and PRDX3, whereas others showed differential expression only later during the therapy (4th vaccination and DTH evaluation)—CLEC2D, CXCR4, SDCBP, SPG21 and VNN2 (Figure 2A).

To confirm this data set, RNA samples from the same patients were assessed for transcriptome profiling (twelve melanoma patients, from MT079 to MT101, Table 1) and were evaluated by qRT-PCR, for expression of CLEC2D, CXCR4, FCGR2A, MS4A7 and CSNK1A1 genes. These genes were selected because of their immunological functional relevance beside their temporal expression profiles. Despite significant variability of data, our results confirmed a pronounced distinct temporal profile between both groups in genes CXCR4, FCGR2A, CLEC2D and CSNK1A1 (Figure 2C), in line with the transcriptome profiling determined by the microarray data (Figure 2A–2B).

**Figure 3:**
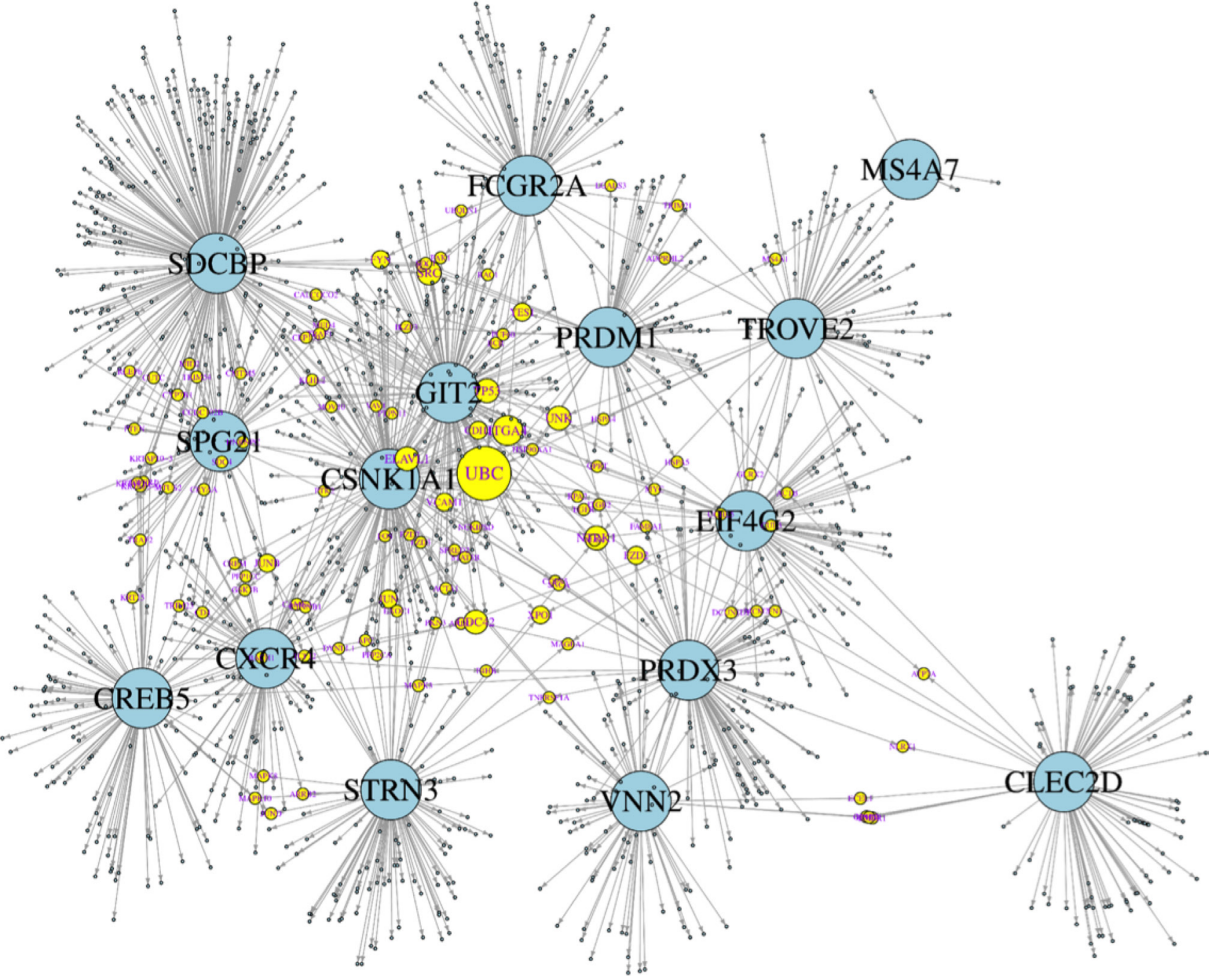
Theoretical network of genetic interactions associated to DC vaccine-induced immune response Fifteen genes significantly regulated during the vaccination period (large, blue circles) were subjected to analyses of direct genetic interactions using STRING and BioGrid platforms. Most interactions were unique for each gene, but a few appeared in two or more lists of the 15 genes attributing to them a connector function (yellow circles; the more of the 15 genes are connected by a connector gene, the larger its diameter) and hence giving rise to a molecular network. For instance, ubiquitin C (UBC) (the connector gene with the most connections) connects 9 out of the 15 genes. The combined STRING and BioGrid genes were entered into “genes.R” available in “R”, and further processed in “igraph”.

Undoubtedly, the genes displaying expression changes are embedded in molecular networks. Therefore, many more genes may be affected by these expression changes and may become tentative targets for pharmacotherapies. Apparently, the 15 genes do not show any functional relationships, owing to the fact that there is a cutoff by the level of statistical significance. In an attempt to search for molecular connections between those genes, each gene was subjected to a nearest neighbor, or cluster analysis using two platforms from internet: STRING (https://string-db.org/) using the setting of a maximum of 50 interactions, and BioGrid (https://thebiogrid.org/), with no limitations of interactions. The platforms list all molecular interactions known from the available literature, which is admittedly a bias. The results from both platforms are listed for each of the 15 genes in Supplementary Table 3. Apparently, the number of interacting genes depends of the knowledge available, and does not necessary reflect the true numbers of biological interactions. In fact, there are variable numbers of genes appearing in both platforms (Supplementary Table 3, highlighted in red), but overall there is little overlap. The combined lists (STRING and BioGrid results) for each gene were entered into “genes.R” available in “R” (https://cran.r-project.org/bin/windows/base/), and turned into a graphical display by using “igraph” (http://igraph.org/r/ a windows binary package). This strategy enabled us to identify molecular targets connecting two or more of the 15 genes, and thus giving rise to a molecular network (Figure 3). For instance, JUN/JUNB appeared in the clusters of CREB5, PRDM1, SDCBP, and STRN3, CDC42 in GIT2, PRDX3, CXCR4, and CSNK1A1, ITGA4 in CSNK1A1, EIF4G2, PRDX3, SDCBP, and FCGR2A and UBC appeared in CSNK1A1, CXCR4, EIF4G2, FCGR2A, PRDX3, SDCBP, SPG21, STRN, and TROVE2, which means that ubiquitin C connects 9 out of the 15 genes. Hypothetically, the gene products (proteins) constituting the network in Figure 3 are the most affected molecular targets in response to the expression changes experimentally discovered during the vaccination period. Hence, the molecular interactions displayed in Figure 3 are parts of a potential “vaccination network” [18]. This network may allow for a better understanding of molecular interactions affected by the 15 genes identified here as differentially regulated by vaccination.

The analysis of gene expression changes during the vaccination protocol for discriminating between patients classified according to their clinical response allowed us to identify 10 potential biomarkers with immunological functions, related to processes such as chemotaxis, endocytosis, phagocytosis, and cytotoxicity—CXCR4, GIT2, FCGR2A, SDCBP and VNN2—as well as activation, differentiation and maturation of myeloid lineage and the lymphoid population—CLEC2D, GIT2, MS4A7, PRDM1, PRDX3, SDCBP and SPG21 (Supplementary Table 2). To further evaluate this potential, we performed Receiver Operating Characteristic (ROC) curve analyses, using the time series values obtained by microarray (Supplementary Table 5) and by qRT-PCR (Table 2) for each gene. It turned out that the PRDM1 and SDCBP genes could distinguish between both groups of patients at early expression time points (ROC curves parameters: AUC = 1, S = 100%, E = 100% and *p* < 0.05), whereas genes CLEC2D, CXCR4, FCGR2A, GIT2, SDCBP, SPG21 and VNN2 showed this discrimination at later expression point (4th vaccination), also with perfect ROC curves parameters (AUC = 1, S = 100%, E = 100%), but with a *p* value at the limit of the significance (*p* = 0.053), suggesting their potential use as predictive or follow-up biomarkers (Supplementary Table 5). Additionally, the MS4A7, PRDM1 and PRDX3 genes also could distinguish between both groups of patients at later expression point (DTH evaluation), with perfect ROC curves parameters, but with a non-significant *p* value (*p* = 0.13) (Supplementary Table 5). qRT-PCR data showed for all the four genes evaluated—CLEC2D, CXCR4, FCGR2A and MS4A7—to be able to discriminate between both groups of patients at least at one point during the treatment, with perfect ROC curves parameters, but with a non-significant *p* value (*p* > 0.12) (Table 2). Suggesting that a higher number of samples evaluated by qRT-PCR could be required to strongly support their value as biomarkers.

**Table 2:** ROC curve analysis assessed by qRT-PCR time-course data

GEN	1st vaccination					2nd vaccination		3rd vaccination					4th vaccination		DTH evaluation				
	AUC	Cut-off	S	E	*p* value	AUC	*p* value	AUC	Cut-off	S	E	*p* value	AUC	*p* value	AUC	Cut-off	S	E	*p* value
CLEC2D	0.75				0.35	0.84	0.11	1	1.46	100	100	0.15	0.75	0.31	1	0.66	100	100	0.12
CXCR4	1	1.48	100	100	0.15	0.68	0.38	0.66				0.61	0.35	0.55	1	0.22	100	100	0.12
FCGR2A	0.87				0.16	0.75	0.24	0.50				1	0.75	0.31	1	0.50	100	100	0.12
MS4A7	0.25				0.35	0.25	0.28	1	0.78	100	100	0.13	0.41	0.73	0.57				0.82

### Protein expression in immune cells from TRIMEL-loaded DCs-treated patients

To investigate if gene expression profiles observed on the transcript level correlated with the protein level, cryopreserved peripheral blood mononuclear cells (PBMCs) obtained from five healthy donors and 19 patients—nine immunological responders and 10 immunological non-responders—were subjected to flow cytometry. From these patients, three had previously been studied by microarrays. Expression data of samples from different vaccination time points were merged for the flow cytometry analysis due to a low availability of samples because of clinical states in individual patients. For that we defined as pre-treatment, samples collected at leukapheresis time, and before 1st and 2nd vaccination; and as post-treatment, samples collected at 3rd, 4th vaccination and at DTH evaluation time point. These criteria were used considering previously reported immunological evaluations for TRIMEL-loaded DC immunotherapy treated patients, which showed a specific cytokine response in PBMC from both DTH+ and DTH- patient subgroups, collected after the 2nd immunization, and after a complete vaccination cycle [15].

To discriminate between lymphocyte and monocyte populations, the side-scatter parameter (SSC), related with cellular complexity, was used combined with the CD45 marker, which is expressed in all hematopoietic cells except in red blood cells (RBCs) and platelets (lymphocyte population, SSC low/CD45+; monocyte population, SSC high/CD45+). Furthermore, CD4+ T-cells (CD3+/CD4+), CD8+ T-cells (CD3+/CD8+), B-cells (CD3–/CD19+), Natural Killer (NK) cells (CD16+/CD56+) and monocyte populations (CD14+) were identified by use of respective differentiation markers. There were a few samples where it was not possible to evaluate all cell populations. In those cases, protein expression was not determined (Supplementary Figure 1).

**Figure 4:**
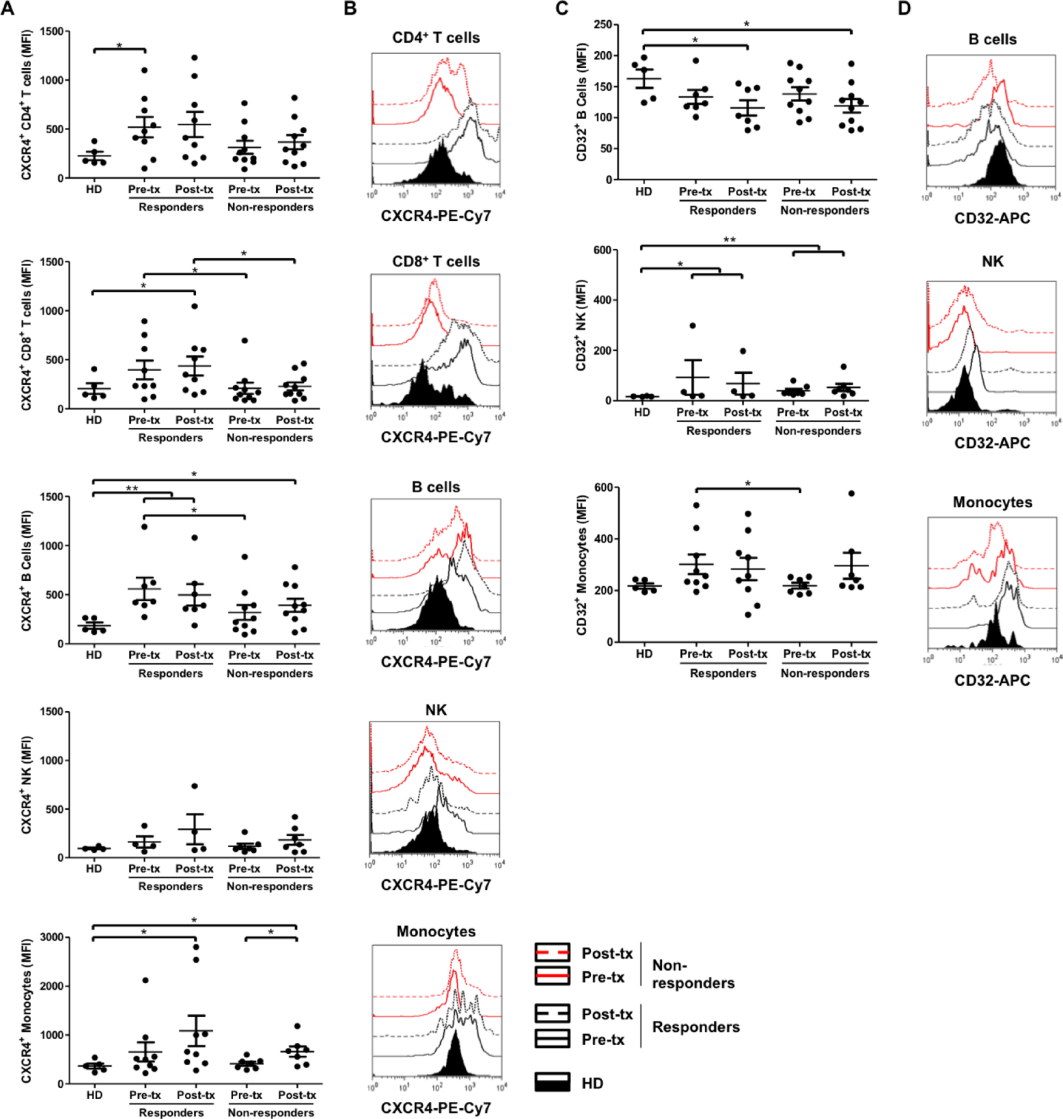
Increased CXCR4 surface expression on CD8^+^ T-cells and B-cells, and CD32 surface expression on monocyte populations in immunological responder compared to non-responder patients (**A**, **C**) Cryopreserved PBMCs, obtained from healthy donors (HD; *n* = 4–5), immunological responder (*n* = 4–9) and non-responder patients (*n* = 7–10), at the beginning (pre-tx) and at the end (post-tx) of TRIMEL-loaded DCs immunization protocol, were analyzed for the CXCR4 (A) and CD32 (C) surface expression by flow cytometry. Each data point represents one patient sample. MFI: mean of fluorescence intensity. Mann-Whitney test; ^*^p < 0.05 ^**^p < 0.01. (**B**, **D**) Representative histograms compare the analysis of one healthy donor (HD), one responder and one non-responder patient to TRIMEL-loaded DCs immunotherapy. CD4^+^ T-cells: CD45^+^/CD3^+^/CD4^+^; CD8^+^ T-cells: CD3^+^/CD8^+^; B-cells: CD3^–^/CD19^+^; NK-cells: CD45^+^/CD16^+^/CD56^+^ and monocyte population: CD45^+^/CD14^+^. Black lines: immunological responder patients; red lines: non-responder patients; continuous lines: pre-tx; dashed lines: post-tx; fill curve: HD; tx, treatment.

Based on the results obtained for gene expression, and taking their immune response related functions into account, the proteins CXCR4, CD32 (which comprises FCGR2A) and CLEC2D were evaluated. Higher CXCR4 protein expression was observed in immunological responder patients compared to healthy donors and immunological non-responder patients, at pre- and post-treatment time points, in all cell types except for NK cells (Figure 4A and 4B). Moreover, non-immunological responders showed a CXCR4 protein expression close to healthy donors, in all evaluated cell types. Significantly higher CXCR4 protein expression was found in immunological responder compared to non-responder patients at pre-treatment, in CD8+ T- and B-cells. These differences were maintained in CD8+ T-cells until post-treatment. Altogether, these results agree with the role of CXCR4 as a potential predictive and/or follow up biomarker. Additionally, significant differences of CXCR4 expression were observed between healthy donors and immunological responder patients at pre-treatment in CD4+ T-cells; at pre- and post-treatment in B-cells; and at post-treatment in CD8+ T-cells and monocyte populations. Also, significant differences of CXCR4 protein expression were obtained between healthy donors and immunological non-responder patients, but only at post-treatment, in B-cells and monocyte populations. Finally, a significant difference was found between pre- and post-treatment time points in monocyte populations from immunological non-responder patients (Figure 4A and 4B).

On the other hand, significantly higher CD32 protein expression in monocyte populations was observed in immunological responder compared to non-responder patients, at pre-treatment time points, which is consistent with its role as a predictive biomarker. Additionally, significant differences of CD32 expression were obtained between healthy donors and immunological responder patients, at pre- and post-treatment in NK-cells; and at post-treatment in B-cells. Similar significant differences were found between healthy donors and non-responder patients (Figure 4C and 4D). Regarding CD32 positive CD4+ and CD8+ T-cells, the average founded was lower than 30% in each population, in agreement to previously reported CD32 expression levels on PBMCs [23–25]. Also, we did not find any statistical significant difference between patients' conditions (data not shown).

Concerning CLEC2D protein expression, despite the variability obtained, significant differences between healthy donors and immunological responder patients, at pre- and post-treatment in CD4+ and CD8+ T-cells, respectively, were observed (Supplementary Figure 2).

Finally, if we consider the expression levels of the three proteins evaluated—CXCR4, CD32 and CLEC2D—in each cell population, and in each patient, a clear profile related to the treatment response is still unresolved.

## DISCUSSION

Several strategies have been designed to identify multiple factors associated with an effective antitumor immune response against melanoma. Until 2010, no randomized clinical trials had provided evidence of improved survival for patients with advanced-stage metastatic melanoma [19]. However, in recent years great improvements in prospective randomized phase III clinical trials have shown prolonged clinical benefits, progression-free survival as well as increased survival rates [19]. In this regard, the present and future of melanoma research will rely on development of immunotherapies and therapies targeted to particular signaling pathways along with identification of predictive biomarkers, which will be critically relevant for treatment optimization. This implies in-depth systematic analysis of major biological mechanisms that affect the clinical efficacy of current therapies.

In this context, we have previously reported a TRIMEL-loaded DC-based immunotherapy capable of extending the survival time in stage IV malignant melanoma patients that respond with a DTH+ immune reaction (median survival: 33 months), compared to patients that do not develop this type of response (DTH-) (median survival: 11 months) [9, 14]. Besides the DTH reaction, other non-detected immunological responses may affect tumor control and impact patient survival. Thus, blood samples obtained from patients with malignant melanoma, before, during and after immunizations with TRIMEL-loaded DCs are highly valuable in this kind of immunotherapy to differentiate between responder and non-responder groups and could become an important source of data with predictive clinical value. Additionally, we could distinguish at the transcriptomic level, during the vaccination protocol, patients who responded positively to the treatment from those who did not respond, correlating with survival time. Results from transcriptome analyses revealed that 66.7% of the regulated genes were related to immune functions, such as chemotaxis, endocytosis, phagocytosis, cytotoxicity processes, as well as activation, differentiation and maturation of myeloid lineage and the lymphoid population. The remaining 33.3% are associated with cell cycle and signal transduction (Figure 2 and Supplementary Table 2).

Interestingly, some of the candidate genes obtained in these expression profiles were the immune-related receptors CXCR4, CD32 and the ligand CLEC2D, which displays relevant immune response functions, such as migration, antibody signaling responses and activation. These tentative targets were further quantified at the protein level within the leukocyte populations of patients with advanced malignant melanoma, which confirmed their capacity to discriminate between immunological responder and non-responder patients to TRIMEL-loaded DC immunotherapy, not only at the end of the immunization protocol, but also before the treatment. We decided to analyze these three markers at protein level for several reasons: 1) mRNA expression levels of these genes were corroborated by qRT-PCR analysis; 2) the ROC curve analyses, for these genes showed a bias between responder and non-responder patients at later expression points, supporting their use as predictive or follow-up biomarkers; 3) the protein product for these genes are expressed in different immune cell populations; and 4) the availability of well characterized antibodies against these genes products for flow cytometry use.

CXCR4 is a chemokine receptor belonging to the G protein-coupled receptor family and is specific for the ligand CXCL12. The CXCR4/CXCL12 complex plays a central role in neutrophil migration, and in the recruitment of leukocyte populations to sites of inflammation [20, 21]. Altered expression of CXCR4 has been reported in several inflammatory disorders and a variety of cancers, where it is associated with proliferation of tumor cells and disease progression [20, 21]. Notably, migration of cytotoxic T lymphocytes (CTLs), derived from two melanoma patients, to autologous tumor cells depended on their expression of CXCR4 [22]. Along these lines, our findings demonstrate a significantly higher expression of CXCR4 in CD8+ T- and B-cells in immunological responder patients from samples obtained at the beginning of the immunization protocol (pre-treatment; leukapheresis, 1st and 2nd vaccination), in comparison with the non-responder group. These differences remain in CD8+ T-cells until the end of the protocol (post-treatment; 3rd, 4th vaccination and DTH evaluation; Figure 4A). This supports the notion that increased CXCR4 expression on lymphocytes of melanoma patients could be further studied as a potential predictive and/or follow-up biomarker.

CD32, a membrane glycoprotein receptor for the IgG crystallizable portion (FCGR), has three isoforms, FCGR2A, B and C. FCGR2A is expressed in all cells of the myeloid lineage, but not in lymphocytes; FCGR2B is expressed in basophils, neutrophils, monocytes, macrophages, DC and B lymphocytes; while FCGR2C is expressed in neutrophils, monocytes and NK cells. CD32 is involved in endocytosis, phagocytosis, antibody-dependent cell-mediated cytotoxicity (ADCC), release of soluble mediators and immunomodulation, making it an interesting target for modulating the immune system. The A and C isoforms have intracellular domains with a pattern of tyrosine-based activation (ITAM), whereas the B isoform contains the inhibitory counterpart (ITIM) [23–25]. Considering that the monocyte population at the beginning of the immunization protocol (pre-treatment; leukapheresis, 1st and 2nd vaccination) in TRIMEL-loaded DC-treated immunological responder patients displayed significantly increased expression of CD32 protein, it would be important to confirm these expression levels for the isoforms by use of specific antibodies, although our gene expression results indicate that these differences are due to the A isoform (FCGR2A) (Figure 2 and 4C). Because this isoform has an ITAM intracellular domain, the activation of endocytosis/phagocytosis processes and the release of soluble mediators, such as cytokines, may be relevant to mount an appropriate immune response for TRIMEL-loaded DC therapy.

On the other hand, CLEC2D protein is expressed in DC, NK-, T- and B-cells, and participates in activation of lymphocyte populations through its interaction with the CD161 receptor, a member of the C-type lectin superfamily, which is found in immature NK cells [26–28]. Since our results on CLEC2D protein expression showed a high variability, we assume that a larger number of patients should be evaluated to find possible differences between patient groups treated with TRIMEL-loaded DC immunotherapy (Supplementary Figure 2).

The present data show that there are consistent differences between immunological responder and non-responder patients to TRIMEL-loaded DC immunotherapy, using CXCR4 and CD32 as tentative biomarkers, although, we cannot discard the dependence of other potential additional factors, as exome mutations, polymorphisms in immune related genes or immunological changes induced by particular tumor mutations in each patient. Moreover, these data include more gene products—CLEC2D, GIT2, MS4A7, PRDM1, PRDX3, SDCBP, SPG21 and VNN2—as potential biological markers that warrant further confirmation, either in the previously analyzed patients or in a follow-up study of new patients. Additionally, protein network analysis provides a more sophisticated insight into possible interactions between the 15 genes differentially regulated by the vaccination process. For instance, JUN/JUNB, CDC42, ITGA4 and UBC appeared connecting from 4 to 9 out of the 15 genes, constituting a theoretically “vaccination network” that included the most affected molecular targets in response to the expression changes experimentally discovered during the vaccination period, giving them a potential as tentative targets for pharmacotherapies. To our knowledge, this is the first identification of candidate genes with predictive value for positive outcomes to a DC-based immunotherapy. Their clinical use as biomarkers depends on further assessments in melanoma patients undergoing TRIMEL-loaded DC therapy.

## MATERIALS AND METHODS

### Patients and TRIMEL-loaded DC vaccination protocol

Peripheral blood samples were collected from 28 advanced melanoma patients (seven stage III and 21 stage IV patients) who had been vaccinated with TRIMEL-loaded DCs and followed up according to a previously described protocol [9]. Briefly, PBMC obtained by leukapheresis, were isolated and adherent monocytes were incubated in serum-free AIM-V medium (therapeutic grade; Invitrogen Corporation, USA) with recombinant human interleukin-4 (rhIL-4; 500 U/mL; US Biologic, USA) and granulocyte macrophage-colony stimulating factor (GM-CSF; 800 U/mL; SheringPlough, Ireland) for 22 h, and then loaded with 100 μg/mL of TRIMEL in the presence of tumor necrosis factor-α (TNF-α; 20 U/mL; US Biologic, USA) to induce DC maturation. After additional 24 h, DCs were recovered and cryopreserved using an automatic freezing system COBE Spectra (Gambro BCT, Inc., USA). Patients were vaccinated intradermally with 1 mL of DCs mixed with aluminum hydroxide (500 μg; J.T. Baker, USA) or Keyhole limpet haemocyanin (KLH; 100 μg; Calbiochem, USA) in the leg or arm closest to intact lymph nodes. The vaccination protocol consisted of four doses injected on days 0, 10, 30, and 50. One month after the end of therapy, patients were assessed for in vivo DTH reactions to the TRIMEL cell lysate, injecting subcutaneously 200 μL of TRIMEL (2 mg/mL) and 100 μL of control antigens KLH (1 mg/mL) or MULTITEST cell-mediated immunity (CMI; Pasteur-Mérieux, France) in saline solutions. Saline solution alone (100 μL) was used as a negative control. A positive reaction was defined as skin erythema or induration ≥5 mm at 48 h after injection. Blood samples were collected from leukapheresis, and before first, second, third, and fourth vaccine injection and also precisely before DTH test were performed.

The study was performed in agreement with the Helsinki Declaration, and approved by the Bioethical Committee for Human Research of the Faculty of Medicine, Universidad de Chile. All patients signed an informed consent. Clinical and pathological characteristics of the patient cohorts including age, gender and disease stage are summarized in Table 1. Twelve patients were analyzed by microarrays and nineteen by flow cytometry (patients MT079, MT080 and MT091 were analyzed with both methods; Table 1).

### RNA isolation

RNA was isolated from 10 mL of patient blood, using the LeukoLOCK™ total RNA Isolation System (Ambion, Inc., USA), according to manufacturer's instructions. Briefly, whole blood was collected in EDTA and passed through the LeukoLOCK filter. The filter captured the total leukocyte population, while plasma, platelets, and RBCs were eliminated. The filters were flushed with 3 mL of PBS to remove residual RBCs and then with 3 mL of RNAlater to stabilize leukocyte RNA. The leukocytes trapped in the filter were lysed and collected in a 15 mL conical tube. Thereafter, RNA was captured from the lysate on RNA binding beads, purified, treated with TURBO DNase and finally eluted.

The quantity of RNA was assessed using the NanoDrop ΝD-1000A spectrophotometer (Thermo Scientific, USA) and RNA quality was evaluated using the Agilent 2100 Bioanalyzer (Agilent Technologies, USA). Only isolates with RNA integrity number (RIN) values equal or higher than 7.0 were used for further studies. RIN was determined using the RIN algorithm of the Agilent 2100 expert software.

### Microarray experiment preparation, hybridization and analysis

Total RNA (0.25 μg or 0.5 μg) was reverse transcribed and amplified with the Illumina TotalPrep kit (Ambion Inc., USA) according to the manufacturer's instructions. Briefly, GPL6884 - Illumina HumanWG-6 v3.0 expression bead chip (Illumina, USA) that entail six arrays per chip (48,803 genes and gene variants derived from RefSeq) were hybridized overnight with biotinylated cDNAs, washed, blocked and fluorescence-labeled using streptavidin-Cy3. Data were obtained and analyzed by the Illumina^®^ Beadarray Reader system, Illumina^®^ BeadStudio Application software and R+ Bioconductor software. Related data have been deposited in NCBI's Gene Expression Omnibus and are accessible through GEO Series accession number GSE106128 (GSM 2830134–2830180). The analysis considered standard normalizations, a cut-off at 20 intensity values and a *p*-value of ≤ 0.01 with respect to the expression level. Subsequently, all genes that displayed *p*-values of > 0.01 or showed expression changes between 0.5- and 2-fold during the treatment in all samples, were eliminated because they were considered too small to be biologically significant. As a result, 13,702 genes were eliminated and 7,628 were included in the final analysis. RNAs obtained from the leukapheresis procedure or from the first sample obtained for the inoculation protocol (patient MT084, 2nd vaccination; patient MT098, 1st vaccination; and patient MT101, 1st vaccination) were used as a fold change reference.

### Semi-quantitative real-time reverse transcriptase polymerase chain reaction (qRT-PCR)

Total RNA (0.5 μg) was reverse-transcribed into cDNA using SuperScript™ III Reverse Transcriptase (Invitrogen, USA) according to manufacturer's instructions. The qRT-PCR was performed with a 1:10 cDNA dilution, using Power SYBR^®^ Green PCR Master Mix (2x) reagent (Applied Biosystems, USA) or ABsolute SYBR Green ROX Mix (2x) (Thermo Scientific, USA). The PCR assay was standardized using an Applied Biosystems 7500 Real-Time PCR System and the SDS 1.3 program or with an ABI Prism 7900HT and the SDS 2.2.2 program (Applied Biosystems, USA). Primers were selected in such a way that annealing temperatures were in a narrow range around 60°C (Supplementary Table 4). PCR was run through 45 cycles. First, 20 “housekeeping” genes were probed, according to the geNorm procedure published by Vandesompele *et al*. in 2002 [29]. Subsequently, RPL27 was chosen as a “housekeeping” gene (Supplementary Table 4). Relative values of gene regulation were calculated by the delta/delta Ct-method.

### Time-course analysis

Genes were selected according to the following rules: (i) The first value in the time series is similar for both clinical responders (DTH+/long time survivors) and non-responder (DTH-) groups; (ii) at least one of the last two values (4th vaccination and DTH evaluation) differ between the patient groups; (iii) the expression dynamics are similar within the groups; (vi) the gene expression shows significant fold changes for the clinical responders, and no significant changes for non-responder patients.

These rules are implemented in form of a set of scores for each gene. To compute these scores, we used the expression dynamics (fold changes with respect to the first value, it means, leukapheresis procedure value. Considering that for patients MT084, MT098 and MT101, there were no values of leukapheresis, the mean of the values obtained for the other evaluated patients was considered as a reference) of the individual genes for each patient gip(t), where i is the gene index, p the index of the patient, and t the time point. R represents the group of clinical responder patients and N the group of non-responder patients. We denote the average expression dynamics for clinical responder patients by *g_p_*(*t*)_*R*_ for non-responder patients by *g_p_*(*t*)_*N*_.

The set of scores includes:

sim: The absolute difference between the average 1st vaccination values for clinical responder and non-responder patients: $\left|{\langle g_{p}^{i}\left(1\right)\rangle }_{p=R}-{\langle g_{p}^{i}\left(1\right)\rangle }_{p=N}\right|$

- diff26: The Euclidean distance between the average time series clinical responder patients ${\langle g_{p}^{i}\left(1\right)\rangle }_{p=R}$ and the average time series of non-responder patients ${\langle g_{p}^{i}\left(1\right)\rangle }_{p=N}$:
  $$\text{dist}\left({\langle g_{p}^{i}\left(1\right)\rangle }_{p=R},{\langle g_{p}^{i}\left(1\right)\rangle }_{p=N}\right)$$
- tscore: For this score we first compute the sign function of the logarithmic fold changes, i.e. 1 if the gene is up-regulated and −1 if the gene is down-regulated. Then sum over the group of clinical responder patients (and non-responder patients) is then computed for the 4th and 5th time points: $\sum_{p=R,t=\left\{4,5\right\}}\text{sign}\left(\log\left(g_{p}^{i}\left(t\right)\right)\right)$

If for the entire group (clinical responder or non-responder) the fold changes are above one, this score assumes a large positive number. If all fold changes are below one, the score is negative. If some fold changes are above and some one, the value of the score is near zero.

SignifR/SignifN: This score gives the number of fold changes above 2 or below 0.5 in all time steps and all patients of the same group (SignifR for clinical responder patients and SignifN for non-responder patients).

absFCR/absFCN: This score represents the sum of the absolute values of logarithmic fold changes, computed separately for clinical responder patients (absFCR) and non-responder patients (absFCRN), e.g. $\sum_{t,p=R}\left|\log\left(g_{p}^{i}\left(t\right)\right)\right|$

A small value of this score means that the fold change is not significant and similar for all time points and patients.

NumSigPos: This score indicates in how many patients of a group (here computed only for clinical responder patients) the fold change of the gene expression is significant (above 2) in at least one time point.

NumSigNeg: This core is the analog to the previous one, with the difference that the significant down-regulations (below 0.5-fold) are considered. By combining NumSigPos and NumSigNeg we identify genes, which show strong up-regulation in all (or at least N) clinical responders, and no significant down-regulation in any patient of the clinical responder group.

Using these scores, we then executed several searches in which a subset of the scores was used to find genes showing specific behavior, in particular:

**Table:** 

1. Genes with similar expression levels for clinical responder and non-responder patients at the first-time point, but large differences between expression levels at later time points.	
Score combination: ***Sim***<0.6 & ***diff26***>5	Genes: VNN2, CXCR4, SDCBP
2. Genes showing upregulated expression with large fold changes for clinical responder patients and down-regulated expression for non-responder patients.	
Score combination: ***tscoreR***>8 & ***tscoreN***<0 & ***Sim***<0.1 & ***SignifR***>5	Genes: LOC648210, GIT2, SPG21, EIF4G2
3. Genes showing strong up-regulation in clinical responders and no change in expression in non-responder patients.	
Score combination: ***tscoreR***>6 & ***tscoreN*** = −1 & ***Sim***<0.1 & ***absFCR***>10.5 & ***absFCN***<2.5	Genes: FCGR2A, CSNK1A1, TROVE2
4. Genes showing a significant up-regulation in 6 out of 7 clinical responder patients, but no significant fold changes in non-responder patients.	
Score combination: ***NumSigPos*** = 6 & ***NumSigNeg*** = −1 & ***SignifN***=0	Genes: PRDM1, CLEC2D
5. Genes showing a significant up-regulation in 5 out of 7 clinical responder patients, but no significant fold changes in non-responder patients.	
Score combination: NumSigPos 5 & NumSigNeg = −1 & SignifN=0	Genes: CREB5, unknown, MS4A7, PRDX3, STRN3

### Flow cytometry

Thawed PBMCs were stained according to the manufacturer's recommendations, with the following antibodies, allophycocyanin (APC)-conjugated anti hCD32 (clone FLI8.26; BD Pharmingen, USA) and anti-hOCIL/CLEC2d (clone 402659; R&D Systems, USA); fluorescein isothiocyanate (FITC)-conjugated anti-hCD14 (clone 61D3) and anti-hCD16 (clone eBioCB16) (eBioscience, USA); phycoerythrin (PE)-conjugated anti-hCD11c (clone 3.9; eBioscience, USA); FITC/PE-conjugated anti-hCD3/CD4 (clone SK7/SK3) and anti-hCD3/CD8 (clone SK7/SK1) (BD Simultest); PE Cy7-conjugated anti-hCD184 (clone 12G5; BD Pharmingen, USA) and peridinin-chlorophyll protein (PerCP)-conjugated anti-hCD19 (clone HIB19) and anti-hCD45 (clone HI30) (BioLegend, USA). To discard dead cells from the analysis, we used Propidium Iodide Staining Reagent Solution (BD Pharmingen, USA) according to manufacturer's instructions. Cells were then incubated with 2 mL of BD FACS Lysing Solution (Becton Dickinson, USA) for 5 min at room temperature, washed and suspended in 500 μL FACS buffer.

Flow cytometry was performed on a FACSCanto II (Becton Dickinson, USA) of the Clinical Hospital of the Universidad de Chile. Analysis of FACS data was performed using the FlowJo software (Tree Star Inc., USA).

### Statistical analysis

Survival curves were calculated using the log-rank (Mantel-Cox) and Gehan-Breslow-Wilcoxon test. Time series analysis was executed using custom software, implemented in Matlab. The evaluation of the diagnostic accuracy of the biomarkers was performed through the construction of ROC curves, by calculating the area under the curve (AUC) value and determining the optimal cut-off points to estimate the highest sensitivity and specificity altogether, as assessed by Youden's Index. Protein network analysis was performed through two platforms from the Internet: STRING (https://string-db.org/) using the basic settings, with a maximum of 50 interactions, and BioGrid (https://thebiogrid.org/), with no limitations of interactions. Statistics were analyzed by GraphPad Prism software (GraphPad Software, Inc., USA). The differences between conditions were evaluated by the nonparametric Mann–Whitney test. All *p*-values are 2-tailed, and a *p*-value of <0.05 was considered statistically significant.

## SUPPLEMENTARY MATERIALS FIGURES AND TABLES









## Abbreviations

Ag: Antigen
ADCC: Antibody-dependent cell-mediated cytotoxicity
APC: Allophycocyanin
AUC: Area under the curve
CTL: Cytotoxic T lymphocyte
DC: Dendritic cell
DTH: Delayed-type hypersensitivity
Fc: Fragment crystallizable
FITC: Fluorescein isothiocyanate
GM-CSF: Granulocyte macrophage-colony stimulating factor
HS: Heat shock
Ig: Immunoglobulin
ITAM: Immunoreceptor tyrosine-based activation motif
ITIM: Immunoreceptor tyrosine-based inhibition motif
KLH: Keyhole limpet haemocyanin
mAbs: monoclonal antibodies
MHC: Major histocompatibility complex
NK: Natural Killer
PBMC: Peripheral blood mononuclear cell
PE: Phycoerythrin
PerCP: Peridinin-chlorophyll protein
qRT-PCR: Quantitative real-time reverse transcriptase PCR
RBCs: Red blood cells
rhIL-4: Recombinant human interleukin-4
ROC: Receiver operating characteristic
RIN: RNA integrity number
SSC: Side-scatter
TAA: Tumor associated antigen
Th: T helper
TLR: Toll-like receptor
TNF-α: Tumor necrosis factor-α
