# Supplementary material for: Molecular signatures associated with tumor-specific immune response in melanoma patients treated with dendritic cell-based immunotherapy
Source: Oncotarget. 2018 Mar 30;9(24):17014–27. doi: 10.18632/oncotarget.24795 (PMC5908302; doi:10.18632/oncotarget.24795)
Supplement: Supplementary file 1 [file oncotarget-09-17014-s001.pdf]

Molecular signatures associated with tumor-specific immune response in melanoma patients treated with dendritic cell-based immunotherapy

SUPPLEMENTARY MATERIALS

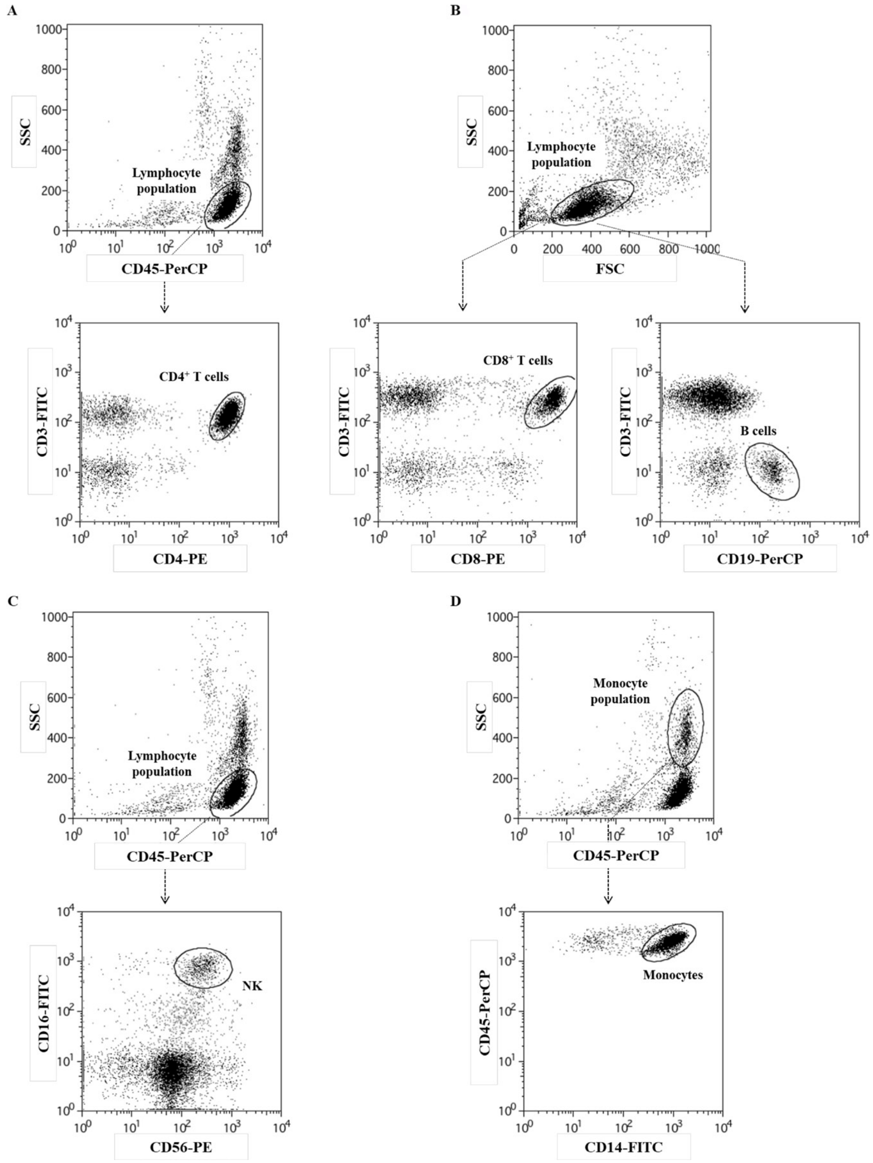

Supplementary Figure 1: Gating strategy for lymphocyte and monocyte populations selection from patients' PBMCs. Representative dot plots of cryopreserved PBMC analyzed by flow cytometry. Selection of lymphocyte populations: (**A**) $CD4^+$ T-cells: $CD45^+/CD3^+/CD4^+$; (**B**) $CD8^+$ T-cells: $CD3^+/CD8^+$, B-cells: $CD3^-/CD19^+$ and (**C**) NK-cells: $CD45^+/CD16^+/CD56^+$. (**D**) Selection of monocyte population: $CD45^+/CD14^+$.

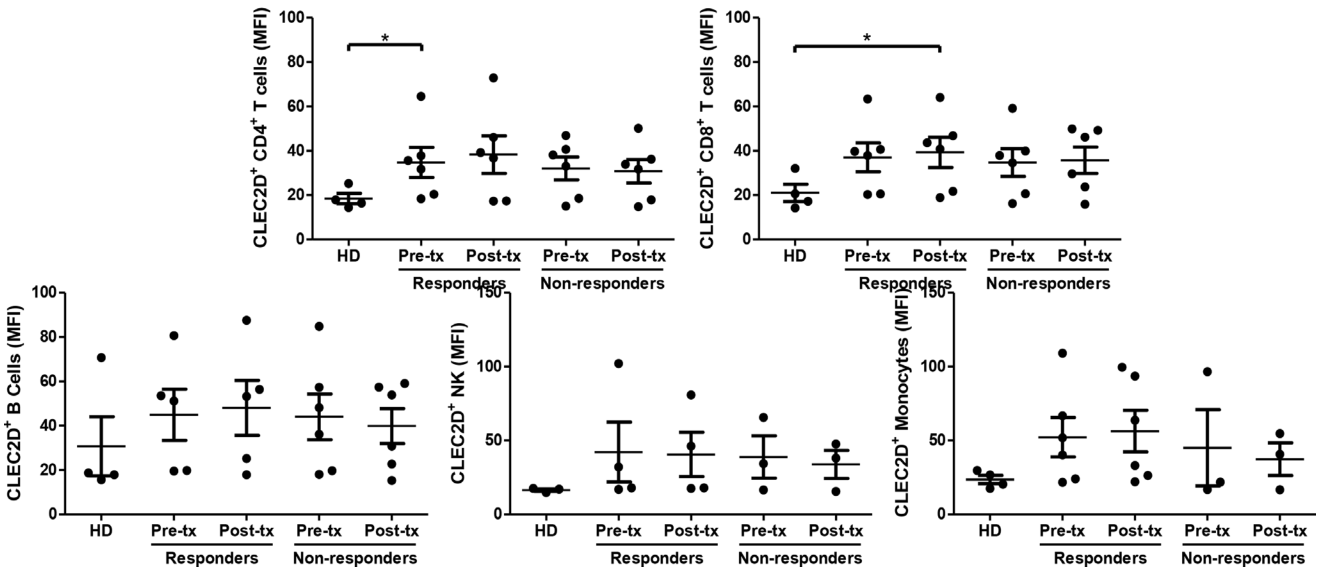

Supplementary Figure 2: CLEC2D surface expression on PBMC from advanced melanoma patients. Cryopreserved PBMCs, obtained from healthy donors (HD; n = 3–4), immunological responder (n = 4–6) and immunological non-responder patients (n = 3–6), at the beginning (pre-tx) and at the end (post-tx) of the TRIMEL-loaded DCs immunization protocol, were analyzed for the CLEC2D surface expression by flow cytometry. Each data point represents one patient's sample. MFI: mean of fluorescence intensity. $CD4^+$ T cells: $CD45^+/CD3^+/CD4^+$; $CD8^+$ T-cells: $CD3^+/CD8^+$; B-cells: $CD3^-/CD19^+$; NK-cells: $CD45^+/CD16^+/CD56^+$ and monocyte population: $CD45^+/CD14^+$. tx, treatment. Mann–Whitney test; $^*p < 0.05$.

Supplementary Table 1: Collected samples from TRIMEL-loaded DCs treated melanoma patients for gene expression analysis

Patient code	Leukapheresis	1st vaccination	2nd vaccination	3rd vaccination	4th vaccination	DTH evaluation	DTH response
MT079	√				√	√	–
MT080	√					√	+
MT083	√	√	√	√	√	√	–
MT084			√	√	√	√	+
MT087	√	√	√				NT
MT089	√	√	√				NT
MT091	√	√	√	√	√		+
MT093	√	√	√	√	√	√	+
MT094	√		√	√	√		NT
MT096	√	√				√	+
MT098		√	√				NT
MT101		√	√	√	√	√	–

Abbreviation: NT, Not Tested.

Supplementary Table 2: Genes differentially expressed in TRIMEL-loaded DCs treated melanoma patient groups. See Supplementary_Table_2

Supplementary Table 3: Predicted functional partners proteins. See Supplementary_Table_3

Supplementary Table 4: qRT-PCR primers

Gen	ID (NCBI)	Sequence (5′→3′)	Orientation
CLEC2D	29121	CAG AGA ACA AGG CCA ACC AT	forward
		CTG GCA CCT TTG TCA TTC AA	reverse
CSNK1A1	1452	TTT GAG GAA GCC CCA GAT TA	forward
		TGC TGT GCT GCT TTC TGC	reverse
CXCR4	7852	ACC GCA TCT GGA GAA CCA	forward
		ATA GTC CCC TGA GCC CAT TT	reverse
FCGR2A	2212	CTG TGA AGG CTG CCC AAT	forward
		GCC GTC AGC TGT TTC ATA GTC	reverse
MS4A7	58475	CTT CAC TGT GCT GGA GCT CTT	forward
		GAC TGG GTC GAG GAA AAT GA	reverse
RPL27	6155	CAT TGA TGA TGG CAC CTC AG	forward
		CTT GGC GAT CTT CTT CTT GC	reverse

Abbreviations: ID, Identification number; NCBI, National Center for Biotechnology Information.

Supplementary Table 5: ROC curve analysis assessed by microarray time-course data. See Supplementary_Table_5

REFERENCES

1. Boles KS, Barten R, Kumaresan PR, Trowsdale J, Mathew PA. Cloning of a new lectin-like receptor expressed on human NK cells. Immunogenetics. 1999; 50:1–7.
2. Zu YL, Maekawa T, Nomura N, Nakata T, Ishii S. Regulation of trans-activating capacity of CRE-BPa by phorbol ester tumor promoter TPA. Oncogene. 1993; 8:2749–2758.
3. Nomura N, Zu YL, Maekawa T, Tabata S, Akiyama T, Ishii S. Isolation and characterization of a novel member of the gene family encoding the cAMP response element-binding protein CRE-BP1. J Biol Chem. 1993; 268:4259–4266.
4. Budd DC, McDonald JE, Tobin AB. Phosphorylation and regulation of a Gq/11-coupled receptor by casein kinase 1alpha. J Biol Chem. 2000; 275:19667–19675.
5. Gonzales ML, Mellman DL, Anderson RA. CKIalpha is associated with and phosphorylates star-PAP and is also required for expression of select star-PAP target messenger RNAs. J Biol Chem. 2008; 283:12665–12673.
6. Chia R, Haddock S, Beilina A, Rudenko IN, Mamais A, Kaganovich A, Li Y, Kumaran R, Nalls MA, Cookson MR. Phosphorylation of LRRK2 by casein kinase 1alpha regulates trans-Golgi clustering via differential interaction with ARHGEF7. Nat Commun. 2014; 5:5827.
7. Liu C, Li Y, Semenov M, Han C, Baeg GH, Tan Y, Zhang Z, Lin X, He X. Control of beta-catenin phosphorylation/degradation by a dual-kinase mechanism. Cell. 2002; 108:837–847.
8. LeBron C, Chen L, Gilkes DM, Chen J. Regulation of MDMX nuclear import and degradation by Chk2 and 14-3-3. EMBO J. 2006; 25:1196–1206.
9. Gilkes DM, Chen L, Chen J. MDMX regulation of p53 response to ribosomal stress. EMBO J. 2006; 25:5614–5625.
10. Wang Y, Sun X, Wu J, Xu BE, Gu C, Wang H, Wang X, Tan F, Peng X, Qiang B, Yuan J, Luo Y. Casein kinase 1alpha interacts with RIP1 and regulates NF-kappaB activation. Biochemistry. 2008; 47:441–448.
11. Inuzuka H, Tseng A, Gao D, Zhai B, Zhang Q, Shaik S, Wan L, Ang XL, Mock C, Yin H, Stommel JM, Gygi S, Lahav G, et al. Phosphorylation by casein kinase I promotes the turnover of the Mdm2 oncoprotein via the SCF(beta-TRCP) ubiquitin ligase. Cancer Cell. 2010; 18:147–159.
12. Kuga T, Kume H, Kawasaki N, Sato M, Adachi J, Shiromizu T, Hoshino I, Nishimori T, Matsubara H, Tomonaga T. A novel mechanism of keratin cytoskeleton organization through casein kinase Ialpha and FAM83H in colorectal cancer. J Cell Sci. 2013; 126:4721–4731.
13. Mines MA, Goodwin JS, Limbird LE, Cui FF, Fan GH. Deubiquitination of CXCR4 by USP14 is critical for both CXCL12-induced CXCR4 degradation and chemotaxis but not ERK ativation. J Biol Chem. 2009; 284:5742–5752.
14. Secchiero P, Zella D, Curreli S, Mirandola P, Capitani S, Gallo RC, Zauli G. Engagement of CD28 modulates CXC chemokine receptor 4 surface expression in both resting and CD3-stimulated CD4+ T cells. J Immunol. 2000; 164:4018–4024.
15. Wegner SA, Ehrenberg PK, Chang G, Dayhoff DE, Sleeker AL, Michael NL. Genomic organization and functional characterization of the chemokine receptor CXCR4, a major entry co-receptor for human immunodeficiency virus type 1. J Biol Chem. 1998; 273:4754–4760.
16. Loetscher M, Geiser T, O'Reilly T, Zwahlen R, Baggiolini M, Moser B. Cloning of a human seven-transmembrane domain receptor, LESTR, that is highly expressed in leukocytes. J Biol Chem. 1994; 269:232–237.
17. Rey M, Vicente-Manzanares M, Viedma F, Yáñez-Mo M, Urzainqui A, Barreiro O, Vázquez J, Sánchez-Madrid F. Cutting edge: association of the motor protein nonmuscle myosin heavy chain-IIA with the C terminus of the chemokine receptor CXCR4 in T lymphocytes. J Immunol. 2002; 169:5410–5414.
18. Cheng ZJ, Zhao J, Sun Y, Hu W, Wu YL, Pei G. beta-arrestin differentially regulates the chemokine receptor CXCR4-mediated signaling and receptor internalization, and this implicates multiple interaction sites between beta-arrestin and CXCR4. J Biol Chem. 2000; 275:2479–2485.
19. Forde S, Tye BJ, Newey SE, Roubelakis M, Smythe J, McGuckin CP, Pettengell R, Watt SM. Endolyn (CD164) modulates the CXCL12-mediated migration of umbilical cord blood CD133+ cells. Blood. 2007; 109:1825–1833.
20. Schwartz V, Lue H, Kraemer S, Korbiel J, Krohn R, Ohl K, Bucala R, Weber C, Bernhagen J. A functional heteromeric MIF receptor formed by CD74 and CXCR4. FEBS Lett. 2009; 583:2749–2757.
21. Pérez-Martinez M, Gordon-Alonso M, Cabrero JR, Barrero-Villar M, Rey M, Mittelbrunn M, Lamana A, Morlino G, Calabia C, Yamazaki H, Shirao T, Vázquez J, González-Amaro R, et al. F-actin-binding protein drebrin regulates CXCR4 recruitment to the immune synapse. J Cell Sci. 2010; 123:1160–1170.
22. El-Haibi CP, Sharma P, Singh R, Gupta P, Taub DD, Singh S, Lillard JW Jr. Differential G protein subunit expression by prostate cancer cells and their interaction with CXCR5. Mol Cancer. 2013; 12:64.
23. Gordon-Alonso M, Rocha-Perugini V, Alvarez S, Ursa A, Izquierdo-Useros N, Martinez-Picado J, Muñoz-Fernández MA, Sánchez-Madrid F. Actin-binding protein drebrin regulates HIV-1-triggered actin polymerization and viral infection. J Biol Chem. 2013; 288:28382–28397.
24. Huang AF, Chen MW, Huang SM, Kao CL, Lai HC, Chan JY. CD164 regulates the tumorigenesis of ovarian surface epithelial cells through the SDF-1alpha/CXCR4 axis. Mol Cancer. 2013; 12:115.
25. Saini V, Marchese A, Majetschak M. CXC chemokine receptor 4 is a cell surface receptor for extracellular ubiquitin. J Biol Chem. 2010; 285:15566–15576.
26. Bhandari D, Robia SL, Marchese A. The E3 ubiquitin ligase atrophin interacting protein 4 binds directly to the chemokine receptor CXCR4 via a novel WW domain-mediated interaction. Mol Biol Cell. 2009; 20:1324–1339.

27. Henis-Korenblit S, Shani G, Sines T, Marash L, Shohat G, Kimchi A. The caspase-cleaved DAP5 protein supports internal ribosome entry site-mediated translation of death proteins. Proc Natl Acad Sci U S A. 2002; 99:5400–5405.
28. Mikami S, Masutani M, Sonenberg N, Yokoyama S, Imataka H. An efficient mammalian cell-free translation system supplemented with translation factors. Protein Expr Purif. 2006; 46:348–357.
29. Guo Z, Neilson LJ, Zhong H, Murray PS, Zanivan S, Zaidel-Bar R. E-cadherin interactome complexity and robustness resolved by quantitative proteomics. Sci Signal. 2014; 7:rs7.
30. Oberer M, Marintchev A, Wagner G. Structural basis for the enhancement of eIF4A helicase activity by eIF4G. Genes Dev. 2005; 19:2212–2223.
31. Lee SH, McCormick F. p97/DAP5 is a ribosome-associated factor that facilitates protein synthesis and cell proliferation by modulating the synthesis of cell cycle proteins. EMBO J. 2006; 25:4008–4019.
32. Khadka S, Vangeloff AD, Zhang C, Siddavatam P, Heaton NS, Wang L, Sengupta R, Sahasrabudhe S, Randall G, Gribskov M, Kuhn RJ, Perera R, LaCount DJ. A physical interaction network of dengue virus and human proteins. Mol Cell Proteomics. 2011; 10:M111012187.
33. Katsogiannou M, Andrieu C, Baylot V, Baudot A, Dusetti NJ, Gayet O, Finetti P, Garrido C, Birnbaum D, Bertucci F, Brun C, Rocchi P. The functional landscape of Hsp27 reveals new cellular processes such as DNA repair and alternative splicing and proposes novel anticancer targets. Mol Cell Proteomics. 2014; 13:3585–3601.
34. Castello A, Fischer B, Eichelbaum K, Horos R, Beckmann BM, Strein C, Davey NE, Humphreys DT, Preiss T, Steinmetz LM, Krijgsveld J, Hentze MW. Insights into RNA biology from an atlas of mammalian mRNA-binding proteins. Cell. 2012; 149:1393–1406.
35. Baltz AG, Munschauer M, Schwanhausser B, Vasile A, Murakawa Y, Schueler M, Youngs N, Penfold-Brown D, Drew K, Milek M, Wyler E, Bonneau R, Selbach M, et al. The mRNA-bound proteome and its global occupancy profile on protein-coding transcripts. Mol Cell. 2012; 46:674–690.
36. Rolland T, Tasan M, Charloteaux B, Pevzner SJ, Zhong Q, Sahni N, Yi S, Lemmens I, Fontanillo C, Mosca R, Kamburov A, Ghiassian SD, Yang X, et al. A proteome-scale map of the human interactome network. Cell. 2014; 159:1212–1226.
37. Frank SR, Adelstein MR, Hansen SH. GIT2 represses Crk- and Rac1-regulated cell spreading and Cdc42-mediated focal adhesion turnover. EMBO J. 2006; 25:1848–1859.
38. Hua KT, Tan CT, Johansson G, Lee JM, Yang PW, Lu HY, Chen CK, Su JL, Chen PB, Wu YL, Chi CC, Kao HJ, Shih HJ, et al. N-alpha-acetyltransferase 10 protein suppresses cancer cell metastasis by binding PIX proteins and inhibiting Cdc42/Rac1 activity. Cancer Cell. 2011; 19:218–231.
39. Wang J, Huo K, Ma L, Tang L, Li D, Huang X, Yuan Y, Li C, Wang W, Guan W, Chen H, Jin C, Wei J, et al. Toward an understanding of the protein interaction network of the human liver. Mol Syst Biol. 2011; 7:536.
40. Dubrovskyi O, Tian X, Poroyko V, Yakubov B, Birukova AA, Birukov KG. Identification of paxillin domains interacting with beta-catenin. FEBS Lett. 2012; 586:2294–2299.
41. Keller AD, Maniatis T. Identification and characterization of a novel repressor of beta-interferon gene expression. Genes Dev. 1991; 5:868–879.
42. Ying HY, Su ST, Hsu PH, Chang CC, Lin IY, Tseng YH, Tsai MD, Shih HM, Lin KI. SUMOylation of Blimp-1 is critical for plasma cell differentiation. EMBO Rep. 2012; 13:631–637.
43. De Simoni S, Goemaere J, Knoops B. Silencing of peroxiredoxin 3 and peroxiredoxin 5 reveals the role of mitochondrial peroxiredoxins in the protection of human neuroblastoma SH-SY5Y cells toward MPP+. Neurosci Lett. 2008; 433:219–224.
44. Tsuji K, Copeland NG, Jenkins NA, Obinata M. Mammalian antioxidant protein complements alkylhydroperoxide reductase (ahpC) mutation in Escherichia coli. Biochem J. 1995; 307:377–381.
45. Yamashita H, Avraham S, Jiang S, London R, Van Veldhoven PP, Subramani S, Rogers RA, Avraham H. Characterization of human and murine PMP20 peroxisomal proteins that exhibit antioxidant activity *in vitro*. J Biol Chem. 1999; 274:29897–29904.
46. Knoops B, Clippe A, Bogard C, Arsalane K, Wattiez R, Hermans C, Duconseille E, Falmagne P, Bernard A. Cloning and characterization of AOEB166, a novel mammalian antioxidant enzyme of the peroxiredoxin family. J Biol Chem. 1999; 274:30451–30458.
47. Cao Z, Bhella D, Lindsay JG. Reconstitution of the mitochondrial PrxIII antioxidant defence pathway: general properties and factors affecting PrxIII activity and oligomeric state. J Mol Biol. 2007; 372:1022–1033.
48. Nagy P, Karton A, Betz A, Peskin AV, Pace P, O'Reilly RJ, Hampton MB, Radom L, Winterbourn CC. Model for the exceptional reactivity of peroxiredoxins 2 and 3 with hydrogen peroxide: a kinetic and computational study. J Biol Chem. 2011; 286:18048–18055.
49. Angeles DC, Gan BH, Onstead L, Zhao Y, Lim KL, Dachsel J, Melrose H, Farrer M, Wszolek ZK, Dickson DW, Tan EK. Mutations in LRRK2 increase phosphorylation of peroxiredoxin 3 exacerbating oxidative stress-induced neuronal death. Hum Mutat. 2011; 32:1390–1397.
50. Feng Y, Liu DQ, Wang Z, Liu Z, Cao HQ, Wang LY, Shi N, Meng XM. AOP-1 interacts with cardiac-specific protein kinase TNNI3K and down-regulates its kinase activity. Biochemistry (Mosc). 2007; 72:1199–1204.
51. Liu L, Yang C, Yuan J, Chen X, Xu J, Wei Y, Yang J, Lin G, Yu L. RPK118, a PX domain-containing protein, interacts

with peroxiredoxin-3 through pseudo-kinase domains. Mol Cells. 2005; 19:39–45.

52. Angeles DC, Ho P, Chua LL, Wang C, Yap YW, Ng C, Zhou Zd, Lim KL, Wszolek ZK, Wang HY, Tan EK. Thiol peroxidases ameliorate LRRK2 mutant-induced mitochondrial and dopaminergic neuronal degeneration in Drosophila. Hum Mol Genet. 2014; 23:3157–3165.

53. Mukhopadhyay SS, Leung KS, Hicks MJ, Hastings PJ, Youssoufian H, Plon SE. Defective mitochondrial peroxiredoxin-3 results in sensitivity to oxidative stress in Fanconi anemia. J Cell Biol. 2006; 175:225–235.

54. Masaki M, Ikeda A, Shiraki E, Oka S, Kawasaki T. Mixed lineage kinase LZK and antioxidant protein-1 activate NF-kappaB synergistically. Eur J Biochem. 2003; 270:76-83.

55. Grootjans JJ, Zimmermann P, Reekmans G, Smets A, Degeest G, Dürr J, David G. Syntenin, a PDZ protein that binds syndecan cytoplasmic domains. Proc Natl Acad Sci U S A. 1997; 94:13683–13688.

56. Koroll M, Rathjen FG, Volkmer H. The neural cell recognition molecule neurofascin interacts with syntenin-1 but not with syntenin-2, both of which reveal self-associating activity. J Biol Chem. 2001; 276:10646–10654.

57. Luyten A, Mortier E, Van Campenhout C, Taelman V, Degeest G, Wuytens G, Lambaerts K, David G, Bellefroid EJ, Zimmermann P. The postsynaptic density 95/disc-large/zona occludens protein syntenin directly interacts with frizzled 7 and supports noncanonical Wnt signaling. Mol Biol Cell. 2008; 19:1594–1604.

58. Wawrzyniak AM, Vermeiren E, Zimmermann P, Ivarsson Y. Extensions of PSD-95/discs large/ZO-1 (PDZ) domains influence lipid binding and membrane targeting of syntenin-1. FEBS Lett. 2012; 586:1445–1451.

59. Geijsen N, Uings IJ, Pals C, Armstrong J, McKinnon M, Raaijmakers JA, Lammers JW, Koenderman L, Coffer PJ. Cytokine-specific transcriptional regulation through an IL-5Ralpha interacting protein. Science. 2001; 293:1136–1138.

60. Egea-Jimenez AL, Gallardo R, Garcia-Pino A, Ivarsson Y, Wawrzyniak AM, Kashyap R, Loris R, Schymkowitz J, Rousseau F, Zimmermann P. Frizzled 7 and PIP2 binding by syntenin PDZ2 domain supports Frizzled 7 trafficking and signalling. Nat Commun. 2016; 7:12101.

61. Baietti MF, Zhang Z, Mortier E, Melchior A, Degeest G, Geeraerts A, Ivarsson Y, Depoortere F, Coomans C, Vermeiren E, Zimmermann P, David G. Syndecan-syntenin-ALIX regulates the biogenesis of exosomes. Nat Cell Biol. 2012; 14:677–685.

62. Hwangbo C, Tae N, Lee S, Kim O, Park OK, Kim J, Kwon SH, Lee JH. Syntenin regulates TGF-beta1-induced Smad activation and the epithelial-to-mesenchymal transition by inhibiting caveolin-mediated TGF-beta type I receptor internalization. Oncogene. 2016; 35:389–401.

63. Sahni N, Yi S, Taipale M, Fuxman Bass JI, Coulombe-Huntington J, Yang F, Peng J, Weile J, Karras GI, Wang Y, Kovács IA, Kamburov A, Krykbaeva I, et al. Widespread macromolecular interaction perturbations in human genetic disorders. Cell. 2015; 161:647–660.

64. Garrido-Urbani S, Garg P, Ghossoub R, Arnold R, Lembo F, Sundell GN, Kim PM, Lopez M, Zimmermann P, Sidhu SS, Ivarsson Y. Proteomic peptide phage display uncovers novel interactions of the PDZ1-2 supramodule of syntenin. FEBS Lett. 2016; 590:3–12.

65. Yachie N, Petsalaki E, Mellor JC, Weile J, Jacob Y, Verby M, Ozturk SB, Li S, Cote AG, Mosca R, Knapp JJ, Ko M, Yu A, et al. Pooled-matrix protein interaction screens using Barcode Fusion Genetics. Mol Syst Biol. 2016; 12:863.

66. Li AL, Li HY, Jin BF, Ye QN, Zhou T, Yu XD, Pan X, Man JH, He K, Yu M, Hu MR, Wang J, Yang SC, et al. A novel eIF5A complex functions as a regulator of p53 and p53-dependent apoptosis. J Biol Chem. 2004; 279:49251–49258.

67. Zeitlmann L, Sirim P, Kremmer E, Kolanus W. Cloning of ACP33 as a novel intracellular ligand of CD4. J Biol Chem. 2001; 276:9123–9132.

68. Rual JF, Venkatesan K, Hao T, Hirozane-Kishikawa T, Dricot A, Li N, Berriz GF, Gibbons FD, Dreze M, Ayivi-Guedehoussou N, Klitgord N, Simon C, Boxem M, et al. Towards a proteome-scale map of the human protein-protein interaction network. Nature. 2005; 437:1173–1178.

69. Yu H, Tardivo L, Tam S, Weiner E, Gebreab F, Fan C, Svrzikapa N, Hirozane-Kishikawa T, Rietman E, Yang X, Sahalie J, Salehi-Ashtiani K, Hao T, et al. Next-generation sequencing to generate interactome datasets. Nat Methods. 2011; 8:478–480.

70. Simonis N, Rual JF, Lemmens I, Boxus M, Hirozane-Kishikawa T, Gatot JS, Dricot A, Hao T, Vertommen D, Legros S, Daakour S, Klitgord N, Martin M, et al. Host-pathogen interactome mapping for HTLV-1 and -2 retroviruses. Retrovirology. 2012; 9:26.

71. Breitman M, Zilberberg A, Caspi M, Rosin-Arbesfeld R. The armadillo repeat domain of the APC tumor suppressor protein interacts with Striatin family members. Biochim Biophys Acta. 2008; 1783:1792–1802.

72. Castets F, Rakitina T, Gaillard S, Moqrich A, Mattei MG, Monneron A. Zinedin, SG2NA, and striatin are calmodulin-binding, WD repeat proteins principally expressed in the brain. J Biol Chem. 2000; 275:19970–19977.

73. Goudreault M, D'Ambrosio LM, Kean MJ, Mullin MJ, Larsen BG, Sanchez A, Chaudhry S, Chen GI, Sicheri F, Nesvizhskii AI, Aebersold R, Raught B, Gingras AC. A PP2A phosphatase high density interaction network identifies a novel striatin-interacting phosphatase and kinase complex linked to the cerebral cavernous malformation 3 (CCM3) protein. Mol Cell Proteomics. 2009; 8:157–171.

74. Couzens AL, Knight JD, Kean MJ, Teo G, Weiss A, Dunham WH, Lin ZY, Bagshaw RD, Sicheri F, Pawson T, Wrana JL, Choi H, Gingras AC. Protein interaction network of the mammalian Hippo pathway reveals mechanisms of kinase-phosphatase interactions. Sci Signal. 2013; 6:rs15.
75. Hauri S, Wepf A, van Drogen A, Varjosalo M, Tapon N, Aebersold R, Gstaiger M. Interaction proteome of human Hippo signaling: modular control of the co-activator YAP1. Mol Syst Biol. 2013; 9:713.
76. Hein MY, Hubner NC, Poser I, Cox J, Nagaraj N, Toyoda Y, Gak IA, Weisswange I, Mansfeld J, Buchholz F, Hyman AA, Mann M. A human interactome in three quantitative dimensions organized by stoichiometries and abundances. Cell. 2015; 163:712–723.
77. Deutscher SL, Harley JB, Keene JD. Molecular analysis of the 60-kDa human Ro ribonucleoprotein. Proc Natl Acad Sci U S A. 1988; 85:9479–9483.
78. Martin F, Malergue F, Pitari G, Philippe JM, Philips S, Chabret C, Granjeaud S, Mattei MG, Mungall AJ, Naquet P, Galland F. Vanin genes are clustered (human 6q22-24 and mouse 10A2B1) and encode isoforms of pantetheinase ectoenzymes. Immunogenetics. 2001; 53:296–306.
