# Supplementary material for: Molecular signatures associated with tumor-specific immune response in melanoma patients treated with dendritic cell-based immunotherapy
Source: Oncotarget. 2018 Mar 30;9(24):17014–27. doi: 10.18632/oncotarget.24795 (PMC5908302; doi:10.18632/oncotarget.24795)
Supplement: Supplementary file 2 [file oncotarget-09-17014-s002.docx]

| **Supplementary Table 2. Genes differentially expressed in TRIMEL-loaded DCs treated melanoma patient groups.** | | | | | |
| --- | --- | --- | --- | --- | --- |
| **Gen** | **ID (NCBI)** | **Full name (NCBI)** | **Function**  **(Gene Ontology)** | **Reference** |  |
| CLEC2D | 29121 | *C-type lectin domain family 2 member D* | ·Transmembrane signaling receptor activity | [1] |  |
| CREB5 | 9586 | *cAMP responsive element binding protein 5* | ·Transcription factor activity, sequence-specific DNA binding  ·Protein binding | [2,3] |  |
| CSNK1A1 | 1452 | *Casein kinase 1 alpha 1* | ·Protein kinase activity  ·Protein serine/threonine kinase activity  ·Protein binding | [[4-12](#_ENREF_32)] |  |
| CXCR4 | 7852 | *C-X-C motif chemokine receptor 4* | ·Co-receptor activity | [[13-26](#_ENREF_21)] |  |
|  |  |  | ·CXC chemokine receptor activity |  |  |
|  |  |  | ·G-protein coupled receptor activity |  |  |
|  |  |  | ·Actin, myosin light chain, protein, ubiquitin and ubiquitin protein ligase binding |  |  |
| EIF4G2 | 1982 | *Eukaryotic translation initiation factor 4 gamma 2* | ·Translation initiation factor activity  ·Translation factor activity, RNA binding  ·Cadherin, protein and RNA binding | [27-35] |  |
| FCGR2A | 2212 | *Fc fragment of IgG receptor IIa* | ·Protein binding | [36] |  |
| GIT2 | 9815 | *GIT ArfGAP 2* | ·Protein binding | [[37-40](#_ENREF_64)] |  |
| MS4A7 | 58475 | *Membrane spanning 4-domains A7* |  |  |  |
| PRDM1 | 639 | *PR/SET domain 1* | ·RNA polymerase II core promoter proximal region sequence-specific DNA binding  ·Transcription factor activity, sequence-specific DNA binding  ·Transcriptional repressor activity, RNA polymerase II core promoter proximal region sequence-specific binding  ·Protein binding | [41,42] |  |
| PRDX3 | 10935 | *Peroxiredoxin 3* | ·Cysteine-type endopeptidase inhibitor activity involved in apoptotic process  ·Alkyl hydroperoxide reductase activity  ·Thioredoxin peroxidase activity | [36, 43-54] |  |
|  |  |  | ·Kinase, protein, protein C-terminus and protein kinase binding |  |  |
| SDCBP | 6386 | *Syndecan binding protein* | ·Cytoskeletal adaptor activity | [29,36,55-70] |  |
|  |  |  | ·Protein heterodimerization activity |  |  |
|  |  |  | ·Cadherin, frizzled*,* identical protein, interleukin-5 receptor, phosphatidylinositol-4,5-bisphosphate, protein, protein N-terminus and syndecan binding |  |  |
| SPG21 | 51324 | *SPG21, maspardin* | ·CD4 receptor and protein binding | [36,63-70] |  |
| STRN3 | 29966 | *Striatin 3* | ·Armadillo repeat domain binding  ·Calmodulin, protein, protein complex and protein phosphatase 2A binding | [[71-76](#_ENREF_98)] |  |
| TROVE2 | 6738 | *TROVE domain family member 2* | ·RNA binding | [77] |  |
| VNN2 | 8875 | *Vanin 2* | ·Pantetheine hydrolase activity | [78] |  |
| Abbreviations: ID, Identification number; NCBI, National Center for Biotechnology Information. | | | | | |
