# Supplementary material for: Molecular signatures associated with tumor-specific immune response in melanoma patients treated with dendritic cell-based immunotherapy
Source: Oncotarget. 2018 Mar 30;9(24):17014–27. doi: 10.18632/oncotarget.24795 (PMC5908302; doi:10.18632/oncotarget.24795)
Supplement: Supplementary file 3 [file oncotarget-09-17014-s003.docx]

| **Supplementary Table 3. Predicted functional partners proteins.** | | | | | | | | | | | | | |
| --- | --- | --- | --- | --- | --- | --- | --- | --- | --- | --- | --- | --- | --- |
| **Protein** | **CLEC2D** | | **CREB5** | | **CSNK1A1** | | | **CXCR4** | | **EIF4G2** | | **FCGR2A** | |
| **Partner protein** | [ABCB6](https://thebiogrid.org/115369/table/homo-sapiens/abcb6.html?sort=official) | [LPHN1](https://thebiogrid.org/116528/table/homo-sapiens/lphn1.html?sort=official) | ADAMTSL4 | KRTAP10-9 | [ACACA](https://thebiogrid.org/106549/table/homo-sapiens/acaca.html?sort=official) | [GAPVD1](https://thebiogrid.org/117568/table/homo-sapiens/gapvd1.html?sort=official) | [RPA3](https://thebiogrid.org/112039/table/homo-sapiens/rpa3.html?sort=official) | ADRBK2 | [NEF](https://thebiogrid.org/1205545/table/human-immunodeficiency-virus-1/nef.html?sort=official) | AP5B1 | MKNK1 | ABI1 | [PITPNA](https://thebiogrid.org/111323/table/homo-sapiens/pitpna.html?sort=official) |
|  | [ABCB8](https://thebiogrid.org/116364/table/homo-sapiens/abcb8.html?sort=official) | [LPHN3](https://thebiogrid.org/116882/table/homo-sapiens/lphn3.html?sort=official) | [AHCYL1](https://thebiogrid.org/115987/table/homo-sapiens/ahcyl1.html?sort=official) | KRTAP3-2 | [ACTA1](https://thebiogrid.org/106573/table/homo-sapiens/acta1.html?sort=official) | [GIGYF1](https://thebiogrid.org/122218/table/homo-sapiens/gigyf1.html?sort=official) | [SEC13](https://thebiogrid.org/112296/table/homo-sapiens/sec13.html?sort=official) | ARR3 | NPY | ARIH1 | MKNK2 | ACTB | PLCG1 |
|  | [ACTB](https://thebiogrid.org/106575/table/homo-sapiens/actb.html?sort=official) | [LRRC8E](https://thebiogrid.org/123131/table/homo-sapiens/lrrc8e.html?sort=official) | AKT1 | KRTAP4-12 | [ADAP1](https://thebiogrid.org/116222/table/homo-sapiens/adap1.html?sort=official) | GLI1 | [SEC16A](https://thebiogrid.org/115247/table/homo-sapiens/sec16a.html?sort=official) | ARRB1 | [NPY1R](https://thebiogrid.org/1205544/table/human-immunodeficiency-virus-1/env.html?sort=official) | ATP7A | [MRPS11](https://thebiogrid.org/122359/table/homo-sapiens/mrps11.html?sort=official) | ACTR2 | PLCG2 |
|  | [ADAM10](https://thebiogrid.org/106616/table/homo-sapiens/adam10.html?sort=official) | [LSR](https://thebiogrid.org/119629/table/homo-sapiens/lsr.html?sort=official) | AKT2 | KRTAP4-2 | [APC](https://thebiogrid.org/106821/table/homo-sapiens/apc.html?sort=official) | GLI2 | [SEC23B](https://thebiogrid.org/115746/table/homo-sapiens/sec23b.html?sort=official) | ARRB2 | NPY2R | BCL2 | NCBP1 | ACTR3 | [PLS1](https://thebiogrid.org/111371/table/homo-sapiens/pls1.html?sort=official) |
|  | [ANTXR1](https://thebiogrid.org/123924/table/homo-sapiens/antxr1.html?sort=official) | [METTL7B](https://thebiogrid.org/128204/table/homo-sapiens/mettl7b.html?sort=official) | AKT3 | KRTAP5-6 | [ARHGAP22](https://thebiogrid.org/121832/table/homo-sapiens/arhgap22.html?sort=official) | [GLI3](https://thebiogrid.org/108999/table/homo-sapiens/gli3.html?sort=official) | [SEC24C](https://thebiogrid.org/230067/table/mus-musculus/sec24c.html?sort=official) | [ATP13A2](https://thebiogrid.org/116973/table/homo-sapiens/atp13a2.html?sort=official) | [NT5E](https://thebiogrid.org/110962/table/homo-sapiens/nt5e.html?sort=official) | CASP1 | [NPM1](https://thebiogrid.org/110929/table/homo-sapiens/npm1.html?sort=official) | [ADPRHL2](https://thebiogrid.org/120276/table/homo-sapiens/adprhl2.html?sort=official) | [PPP3R1](https://thebiogrid.org/111526/table/homo-sapiens/ppp3r1.html?sort=official) |
|  | [ARV1](https://thebiogrid.org/122307/table/homo-sapiens/arv1.html?sort=official) | [METTL9](https://thebiogrid.org/119297/table/homo-sapiens/mettl9.html?sort=official) | APPBP2 | KRTAP5-9 | [ATOH1](https://thebiogrid.org/106964/table/homo-sapiens/atoh1.html?sort=official) | [GNB2](https://thebiogrid.org/109045/table/homo-sapiens/gnb2.html?sort=official) | SFN | [B2M](https://thebiogrid.org/107044/table/homo-sapiens/b2m.html?sort=official) | [NTRK3](https://thebiogrid.org/110971/table/homo-sapiens/ntrk3.html?sort=official) | CASP10 | [NTRK1](https://thebiogrid.org/110969/table/homo-sapiens/ntrk1.html?sort=official) | [AGFG1](https://thebiogrid.org/109503/table/homo-sapiens/agfg1.html?sort=official) | [PROSC](https://thebiogrid.org/116381/table/homo-sapiens/prosc.html?sort=official) |
|  | [ATF6](https://thebiogrid.org/116586/table/homo-sapiens/atf6.html?sort=official) | [MFSD3](https://thebiogrid.org/125254/table/homo-sapiens/mfsd3.html?sort=official) | ARIH1 | KRTAP9-2 | [AXIN1](https://thebiogrid.org/113909/table/homo-sapiens/axin1.html?sort=official) | GSK3B | [SH3KBP1](https://thebiogrid.org/119029/table/homo-sapiens/sh3kbp1.html?sort=official) | [B4GAT1](https://thebiogrid.org/116229/table/homo-sapiens/b4gat1.html?sort=official) | [OSTM1](https://thebiogrid.org/118788/table/homo-sapiens/ostm1.html?sort=official) | CASP2 | POLDIP3 | APCS | [PRTFDC1](https://thebiogrid.org/121276/table/homo-sapiens/prtfdc1.html?sort=official) |
|  | [ATF6B](https://thebiogrid.org/107778/table/homo-sapiens/atf6b.html?sort=official) | [NARS2](https://thebiogrid.org/122846/table/homo-sapiens/nars2.html?sort=official) | ATF1 | KRTAP9-4 | AXIN2 | [GTSE1](https://thebiogrid.org/119580/table/homo-sapiens/gtse1.html?sort=official) | [SHQ1](https://thebiogrid.org/34887/table/saccharomyces-cerevisiae/shq1.html?sort=official) | [CAV1](https://thebiogrid.org/107305/table/homo-sapiens/cav1.html?sort=official) | [P4HB](https://thebiogrid.org/111073/table/homo-sapiens/p4hb.html?sort=official) | CASP3 | PTBP1 | [APEX1](https://thebiogrid.org/106825/table/homo-sapiens/apex1.html?sort=official) | [PTPN11](https://thebiogrid.org/111745/table/homo-sapiens/ptpn11.html?sort=official) |
|  | [ATP12A](https://thebiogrid.org/106969/table/homo-sapiens/atp12a.html?sort=official) | [ND5](https://thebiogrid.org/110636/table/homo-sapiens/nd5.html?sort=official) | ATF2 | LGALS13 | [BACH1](https://thebiogrid.org/107047/table/homo-sapiens/bach1.html?sort=official) | [HERC1](https://thebiogrid.org/114439/table/homo-sapiens/herc1.html?sort=official) | [SIK2](https://thebiogrid.org/116840/table/homo-sapiens/sik2.html?sort=official) | [CCDC107](https://thebiogrid.org/128462/table/homo-sapiens/ccdc107.html?sort=official) | PMCH | CASP4 | PTBP2 | ARPC1A | RAC1 |
|  | [ATP7A](https://thebiogrid.org/107020/table/homo-sapiens/atp7a.html?sort=official) | [NETO2](https://thebiogrid.org/123589/table/homo-sapiens/neto2.html?sort=official) | ATF4 | LGR4 | [BCL10](https://thebiogrid.org/114429/table/homo-sapiens/bcl10.html?sort=official) | [HMGB1](https://thebiogrid.org/109389/table/homo-sapiens/hmgb1.html?sort=official) | [SMO](https://thebiogrid.org/59452/table/drosophila-melanogaster/smo.html?sort=official) | CCL11 | [PPBP](https://thebiogrid.org/119232/table/homo-sapiens/golt1b.html?sort=official) | CASP5 | [QPRT](https://thebiogrid.org/117035/table/homo-sapiens/qprt.html?sort=official) | ARPC2 | [SCRN1](https://thebiogrid.org/115145/table/homo-sapiens/scrn1.html?sort=official) |
|  | [ATP7B](https://thebiogrid.org/107022/table/homo-sapiens/atp7b.html?sort=official) | [NLRX1](https://thebiogrid.org/122796/table/homo-sapiens/nlrx1.html?sort=official) | ATF7 | MAP9 | [BCR](https://thebiogrid.org/107083/table/homo-sapiens/bcr.html?sort=official) | [HMGB2](https://thebiogrid.org/109391/table/homo-sapiens/hmgb2.html?sort=official) | [SNCA](https://thebiogrid.org/112506/table/homo-sapiens/snca.html?sort=official) | CCL19 | [PTK2](https://thebiogrid.org/111719/table/homo-sapiens/ptk2.html?sort=official) | CASP6 | RAB7A | ARPC3 | SRC |
|  | [ATP8B2](https://thebiogrid.org/121445/table/homo-sapiens/atp8b2.html?sort=official) | [NXPE3](https://thebiogrid.org/124878/table/homo-sapiens/nxpe3.html?sort=official) | BACH1 | MAPK1 | [BTRC](https://thebiogrid.org/114457/table/homo-sapiens/btrc.html?sort=official) | [HMMR](https://thebiogrid.org/109404/table/homo-sapiens/hmmr.html?sort=official) | [SNX22](https://thebiogrid.org/122945/table/homo-sapiens/snx22.html?sort=official) | CCL21 | PTPN11 | CASP7 | [RNF126](https://thebiogrid.org/120790/table/homo-sapiens/rnf126.html?sort=official) | ARPC5 | SYK |
|  | [BACE2](https://thebiogrid.org/117353/table/homo-sapiens/bace2.html?sort=official) | [POMGNT1](https://thebiogrid.org/120763/table/homo-sapiens/pomgnt1.html?sort=official) | BATF3 | MAPK10 | BYSL | [HNRNPC](https://thebiogrid.org/109424/table/homo-sapiens/hnrnpc.html?sort=official) | [SNX24](https://thebiogrid.org/118791/table/homo-sapiens/snx24.html?sort=official) | CCL25 | [PTPN6](https://thebiogrid.org/111742/table/homo-sapiens/ptpn6.html?sort=official) | CASP8 | [RPA2](https://thebiogrid.org/112038/table/homo-sapiens/rpa2.html?sort=official) | [ARPIN](https://thebiogrid.org/131507/table/homo-sapiens/arpin.html?sort=official) | [TES](https://thebiogrid.org/117572/table/homo-sapiens/tes.html?sort=official) |
|  | [C17orf75](https://thebiogrid.org/122087/table/homo-sapiens/c17orf75.html?sort=official) | [PTPRU](https://thebiogrid.org/115386/table/homo-sapiens/ptpru.html?sort=official) | BRCA1 | MAPK12 | [C1orf111](https://thebiogrid.org/129933/table/homo-sapiens/c1orf111.html?sort=official) | [HSP90AA1](https://thebiogrid.org/109552/table/homo-sapiens/hsp90aa1.html?sort=official) | SPECC1 | CCL27 | [SDC4](https://thebiogrid.org/127873/table/homo-sapiens/ifnlr1.html?sort=official) | CASP9 | [SKIL](https://thebiogrid.org/112389/table/homo-sapiens/skil.html?sort=official) | BAIAP2 | TLR4 |
|  | [CANX](https://thebiogrid.org/107271/table/homo-sapiens/canx.html?sort=official) | [S1PR1](https://thebiogrid.org/108225/table/homo-sapiens/s1pr1.html?sort=official) | CAMK2A | MAPK14 | [CARD11](https://thebiogrid.org/124073/table/homo-sapiens/card11.html?sort=official) | [HSPA4](https://thebiogrid.org/109540/table/homo-sapiens/hspa4.html?sort=official) | [SQSTM1](https://thebiogrid.org/114397/table/homo-sapiens/sqstm1.html?sort=official) | CCL5 | [SLC1A1](https://thebiogrid.org/112396/table/homo-sapiens/slc1a1.html?sort=official) | [CDH1](https://thebiogrid.org/107434/table/homo-sapiens/cdh1.html?sort=official) | SLIRP | BCR | [TRIM21](https://thebiogrid.org/112615/table/homo-sapiens/trim21.html?sort=official) |
|  | [CHPT1](https://thebiogrid.org/121309/table/homo-sapiens/chpt1.html?sort=official) | [SEMA4F](https://thebiogrid.org/115764/table/homo-sapiens/sema4f.html?sort=official) | CAMK2B | MAPK3 | [CBX1](https://thebiogrid.org/198534/table/mus-musculus/cbx1.html?sort=official) | [ITGA4](https://thebiogrid.org/109883/table/homo-sapiens/itga4.html?sort=official) | [SSMEM1](https://thebiogrid.org/126451/table/homo-sapiens/ssmem1.html?sort=official) | CCR5 | [SLC3A2](https://thebiogrid.org/112411/table/homo-sapiens/slc3a2.html?sort=official) | CEP63 | TP53 | [BID](https://thebiogrid.org/107106/table/homo-sapiens/bid.html?sort=official) | UBC |
|  | CLEC1A | [SLC19A2](https://thebiogrid.org/115811/table/homo-sapiens/slc19a2.html?sort=official) | CAMK2D | MAPK8 | [CBY1](https://thebiogrid.org/117311/table/homo-sapiens/cby1.html?sort=official) | [KCTD3](https://thebiogrid.org/119320/table/homo-sapiens/kctd3.html?sort=official) | [SYT9](https://thebiogrid.org/126802/table/homo-sapiens/syt9.html?sort=official) | [CCR5](https://thebiogrid.org/107639/table/homo-sapiens/ccr5.html?sort=official) | [SOCS3](https://thebiogrid.org/114488/table/homo-sapiens/socs3.html?sort=official) | [CYP1A1](https://thebiogrid.org/107923/table/homo-sapiens/cyp1a1.html?sort=official) | UBC | BLK | [UBE2D2](https://thebiogrid.org/113170/table/homo-sapiens/ube2d2.html?sort=official) |
|  | CLEC1B | [SLC22A18](https://thebiogrid.org/111044/table/homo-sapiens/slc22a18.html?sort=official) | CAMK2G | MDFI | [CDC25A](https://thebiogrid.org/107428/table/homo-sapiens/cdc25a.html?sort=official) | [KIF13B](https://thebiogrid.org/116895/table/homo-sapiens/kif13b.html?sort=official) | [TEX264](https://thebiogrid.org/119503/table/homo-sapiens/tex264.html?sort=official) | [CD4](https://thebiogrid.org/107358/table/homo-sapiens/cd4.html?sort=official) | [SST](https://thebiogrid.org/123747/table/homo-sapiens/itch.html?sort=official) | DAP | UBE2L6 | [BLVRB](https://thebiogrid.org/107114/table/homo-sapiens/blvrb.html?sort=official) | [UBQLN1](https://thebiogrid.org/119007/table/homo-sapiens/ubqln1.html?sort=official) |
|  | CLECL1 | [SLC22A23](https://thebiogrid.org/121960/table/homo-sapiens/slc22a23.html?sort=official) | CAMK4 | MGAT5B | CDC42 | [KPNA2](https://thebiogrid.org/110036/table/homo-sapiens/kpna2.html?sort=official) | [TIAM1](https://thebiogrid.org/112930/table/homo-sapiens/tiam1.html?sort=official) | [CD79B](https://thebiogrid.org/107412/table/homo-sapiens/cd79b.html?sort=official) | [ST13](https://thebiogrid.org/112644/table/homo-sapiens/st13.html?sort=official) | [DCUN1D1](https://thebiogrid.org/119920/table/homo-sapiens/dcun1d1.html?sort=official) | [UNK](https://thebiogrid.org/124536/table/homo-sapiens/unk.html?sort=official) | [CBL](https://thebiogrid.org/107315/table/homo-sapiens/cbl.html?sort=official) | VAV1 |
|  | [CNTNAP3](https://thebiogrid.org/123011/table/homo-sapiens/cntnap3.html?sort=official) | [SLC25A20](https://thebiogrid.org/107241/table/homo-sapiens/slc25a20.html?sort=official) | [CEBPG](https://thebiogrid.org/107483/table/homo-sapiens/cebpg.html?sort=official) | MLLT6 | [CDH1](https://thebiogrid.org/107434/table/homo-sapiens/cdh1.html?sort=official) | [KSR1](https://thebiogrid.org/114371/table/homo-sapiens/ksr1.html?sort=official) | [TNFRSF1B](https://thebiogrid.org/112987/table/homo-sapiens/tnfrsf1b.html?sort=official) | CDC42 | [STAM](https://thebiogrid.org/113722/table/homo-sapiens/stam.html?sort=official) | [EGFR](https://thebiogrid.org/108276/table/homo-sapiens/egfr.html?sort=official) | [XPO1](https://thebiogrid.org/113348/table/homo-sapiens/xpo1.html?sort=official) | CD3G | VAV2 |
|  | [COLEC12](https://thebiogrid.org/123353/table/homo-sapiens/colec12.html?sort=official) | [SLC25A6](https://thebiogrid.org/106790/table/homo-sapiens/slc25a6.html?sort=official) | CHD5 | MTUS2 | [CEP128](https://thebiogrid.org/126920/table/homo-sapiens/cep128.html?sort=official) | [LRP5](https://thebiogrid.org/110220/table/homo-sapiens/lrp5.html?sort=official) | [TOM20](https://thebiogrid.org/33324/table/saccharomyces-cerevisiae/tom20.html?sort=official) | CHRM2 | [STAT1](https://thebiogrid.org/109920/table/homo-sapiens/jak2.html?sort=official) | EIF1 |  | [CHMP4A](https://thebiogrid.org/118852/table/homo-sapiens/chmp4a.html?sort=official) | VAV3 |
|  | [DNAJC1](https://thebiogrid.org/122105/table/homo-sapiens/dnajc1.html?sort=official) | [SLC29A1](https://thebiogrid.org/108344/table/homo-sapiens/slc29a1.html?sort=official) | COL8A1 | NOTCH2ML | [CEP135](https://thebiogrid.org/115018/table/homo-sapiens/cep135.html?sort=official) | [LRP6](https://thebiogrid.org/110219/table/homo-sapiens/lrp6.html?sort=official) | [TP53](https://thebiogrid.org/113010/table/homo-sapiens/tp53.html?sort=official) | CHRM4 | STAT2 | EIF2A |  | CRK | WASF2 |
|  | [DYNC2LI1](https://thebiogrid.org/119644/table/homo-sapiens/dync2li1.html?sort=official) | [SLC2A8](https://thebiogrid.org/119013/table/homo-sapiens/slc2a8.html?sort=official) | CREB1 | PPP1CA | [CEP170](https://thebiogrid.org/115193/table/homo-sapiens/cep170.html?sort=official) | [MALT1](https://thebiogrid.org/116098/table/homo-sapiens/malt1.html?sort=official) | TSR1 | CXCL1 | [STAT3](https://thebiogrid.org/109921/table/homo-sapiens/jak3.html?sort=official) | EIF2S1 |  | CRP | YES1 |
|  | [ECEL1](https://thebiogrid.org/114820/table/homo-sapiens/ecel1.html?sort=official) | [SLC39A1](https://thebiogrid.org/118050/table/homo-sapiens/slc39a1.html?sort=official) | [CREB5](https://thebiogrid.org/114954/table/homo-sapiens/creb5.html?sort=official) | PPP1CB | [CGN](https://thebiogrid.org/121589/table/homo-sapiens/cgn.html?sort=official) | MAP3K1 | [TTC9C](https://thebiogrid.org/129507/table/homo-sapiens/ttc9c.html?sort=official) | CXCL10 | [STAT5B](https://thebiogrid.org/200907/table/mus-musculus/kcnk1.html?sort=official) | EIF2S2 |  | [CRYAB](https://thebiogrid.org/107800/table/homo-sapiens/cryab.html?sort=official) |  |
|  | [EVI5L](https://thebiogrid.org/125449/table/homo-sapiens/evi5l.html?sort=official) | [SLC39A11](https://thebiogrid.org/128379/table/homo-sapiens/slc39a11.html?sort=official) | CREBBP | PPP1CC | [CHRM3](https://thebiogrid.org/107553/table/homo-sapiens/chrm3.html?sort=official) | [MAPT](https://thebiogrid.org/110308/table/homo-sapiens/mapt.html?sort=official) | TUT1 | CXCL11 | [TMEM171](https://thebiogrid.org/126392/table/homo-sapiens/tmem171.html?sort=official) | EIF2S3 |  | CYFIP2 |  |
|  | [FAM189B](https://thebiogrid.org/115938/table/homo-sapiens/fam189b.html?sort=official) | [SLC39A3](https://thebiogrid.org/119011/table/homo-sapiens/slc39a3.html?sort=official) | CREM | PPP2R2D | [CLTC](https://thebiogrid.org/107623/table/homo-sapiens/cltc.html?sort=official) | [MDM2](https://thebiogrid.org/110358/table/homo-sapiens/mdm2.html?sort=official) | UBC | CXCL12 | [TMEM63B](https://thebiogrid.org/120640/table/homo-sapiens/tmem63b.html?sort=official) | EIF3A |  | DOCK1 |  |
|  | [FAM3C](https://thebiogrid.org/115712/table/homo-sapiens/fam3c.html?sort=official) | [SLC47A1](https://thebiogrid.org/120535/table/homo-sapiens/slc47a1.html?sort=official) | CRTC2 | PRKACA | [COPS5](https://thebiogrid.org/116183/table/homo-sapiens/cops5.html?sort=official) | [MDM4](https://thebiogrid.org/110359/table/homo-sapiens/mdm4.html?sort=official) | [UNC45A](https://thebiogrid.org/120986/table/homo-sapiens/unc45a.html?sort=official) | CXCL13 | [TMEM9](https://thebiogrid.org/128927/table/homo-sapiens/tmem9.html?sort=official) | [EIF3E](https://thebiogrid.org/200775/table/mus-musculus/eif3e.html?sort=official) |  | [EEA1](https://thebiogrid.org/113999/table/homo-sapiens/eea1.html?sort=official) |  |
|  | [FAM69A](https://thebiogrid.org/132793/table/homo-sapiens/fam69a.html?sort=official) | [SLC7A3](https://thebiogrid.org/124329/table/homo-sapiens/slc7a3.html?sort=official) | CSNK2A2 | PRKACB | [CPSF6](https://thebiogrid.org/116238/table/homo-sapiens/cpsf6.html?sort=official) | [MYO18A](https://thebiogrid.org/134384/table/homo-sapiens/myo18a.html?sort=official) | [USO1](https://thebiogrid.org/207788/table/mus-musculus/uso1.html?sort=official) | [CXCL14](https://thebiogrid.org/106666/table/homo-sapiens/adrbk2.html?sort=official) | [UBC](https://thebiogrid.org/1205545/table/human-immunodeficiency-virus-1/nef.html?sort=official) | EIF3F |  | [EFHD2](https://thebiogrid.org/122597/table/homo-sapiens/efhd2.html?sort=official) |  |
|  | [FAM8A1](https://thebiogrid.org/119541/table/homo-sapiens/fam8a1.html?sort=official) | [SPPL2B](https://thebiogrid.org/121255/table/homo-sapiens/sppl2b.html?sort=official) | EFEMP2 | PRKACG | [CRBN](https://thebiogrid.org/119360/table/homo-sapiens/crbn.html?sort=official) | [MZT2A](https://thebiogrid.org/576069/table/homo-sapiens/mzt2a.html?sort=official) | [USP34](https://thebiogrid.org/115085/table/homo-sapiens/usp34.html?sort=official) | CXCL9 | [USP14](https://thebiogrid.org/114551/table/homo-sapiens/usp14.html?sort=official) | EIF3H |  | ELMO1 |  |
|  | [FANCL](https://thebiogrid.org/120429/table/homo-sapiens/fancl.html?sort=official) | [ST6GALNAC3](https://thebiogrid.org/129166/table/homo-sapiens/st6galnac3.html?sort=official) | EP300 | PRKCA | CREM | [MZT2B](https://thebiogrid.org/123111/table/homo-sapiens/mzt2b.html?sort=official) | [VCAM1](https://thebiogrid.org/113255/table/homo-sapiens/vcam1.html?sort=official) | CXCR2 | VAV1 | [EIF3I](https://thebiogrid.org/207720/table/mus-musculus/eif3i.html?sort=official) |  | ELMO2 |  |
|  | [FASTKD1](https://thebiogrid.org/122800/table/homo-sapiens/fastkd1.html?sort=official) | [STARD3](https://thebiogrid.org/116148/table/homo-sapiens/stard3.html?sort=official) | EWSR1 | PRKCB | [CSNK1D](https://thebiogrid.org/107837/table/homo-sapiens/csnk1d.html?sort=official) | [NCKIPSD](https://thebiogrid.org/119583/table/homo-sapiens/nckipsd.html?sort=official) | [WEE1](https://thebiogrid.org/113303/table/homo-sapiens/wee1.html?sort=official) | CXCR5 | [VIPR2](https://thebiogrid.org/113275/table/homo-sapiens/vipr2.html?sort=official) | EIF3J |  | [FAHD2A](https://thebiogrid.org/119218/table/homo-sapiens/fahd2a.html?sort=official) |  |
|  | [FASTKD3](https://thebiogrid.org/122523/table/homo-sapiens/fastkd3.html?sort=official) | [SUSD1](https://thebiogrid.org/122169/table/homo-sapiens/susd1.html?sort=official) | FOS | PRKCG | [CSNK1E](https://thebiogrid.org/107838/table/homo-sapiens/csnk1e.html?sort=official) | [NFATC1](https://thebiogrid.org/110845/table/homo-sapiens/nfatc1.html?sort=official) | [XPO1](https://thebiogrid.org/113348/table/homo-sapiens/xpo1.html?sort=official) | [DRD2](https://thebiogrid.org/107044/table/homo-sapiens/b2m.html?sort=official) |  | EIF4A1 |  | [FCGR2B](https://thebiogrid.org/108507/table/homo-sapiens/fcgr2b.html?sort=official) |  |
|  | [FITM2](https://thebiogrid.org/126125/table/homo-sapiens/fitm2.html?sort=official) | [SV2A](https://thebiogrid.org/115229/table/homo-sapiens/sv2a.html?sort=official) | FOSL1 | PRKG1 | CTNNB1 | NFATC2 | [XRCC6](https://thebiogrid.org/108822/table/homo-sapiens/xrcc6.html?sort=official) | [ENV](https://thebiogrid.org/1205544/table/human-immunodeficiency-virus-1/env.html?sort=official) |  | EIF4A2 |  | [FCGR2C](https://thebiogrid.org/114556/table/homo-sapiens/fcgr2c.html?sort=official) |  |
|  | [FNDC3A](https://thebiogrid.org/116530/table/homo-sapiens/fndc3a.html?sort=official) | [TM9SF1](https://thebiogrid.org/115799/table/homo-sapiens/tm9sf1.html?sort=official) | FOSL2 | PRKG2 | [DBNDD1](https://thebiogrid.org/122478/table/homo-sapiens/dbndd1.html?sort=official) | [NFATC3](https://thebiogrid.org/110848/table/homo-sapiens/nfatc3.html?sort=official) | YWHAB | [F2](https://thebiogrid.org/107305/table/homo-sapiens/cav1.html?sort=official) |  | EIF4A3 |  | FCGR3A |  |
|  | [FRMD5](https://thebiogrid.org/124407/table/homo-sapiens/frmd5.html?sort=official) | [TMEM120A](https://thebiogrid.org/123777/table/homo-sapiens/tmem120a.html?sort=official) | GSK3B | PRKX | [DBR1](https://thebiogrid.org/119344/table/homo-sapiens/dbr1.html?sort=official) | [NFKBIA](https://thebiogrid.org/110859/table/homo-sapiens/nfkbia.html?sort=official) | [YWHAE](https://thebiogrid.org/113363/table/homo-sapiens/ywhae.html?sort=official) | [GCNT3](https://thebiogrid.org/114671/table/homo-sapiens/gcnt3.html?sort=official) |  | EIF4B |  | FCGRT |  |
|  | [FZD2](https://thebiogrid.org/108811/table/homo-sapiens/fzd2.html?sort=official) | [TMEM120B](https://thebiogrid.org/126849/table/homo-sapiens/tmem120b.html?sort=official) | HSF1 | RBPMS | [DENND4C](https://thebiogrid.org/120799/table/homo-sapiens/dennd4c.html?sort=official) | [NIN](https://thebiogrid.org/119372/table/homo-sapiens/nin.html?sort=official) | YWHAG | GNA13 |  | EIF4E |  | FGR |  |
|  | [GALNT11](https://thebiogrid.org/121988/table/homo-sapiens/galnt11.html?sort=official) | [TMEM184B](https://thebiogrid.org/117357/table/homo-sapiens/tmem184b.html?sort=official) | HSF2 | RGS20 | DVL1 | [NTRK1](https://thebiogrid.org/110969/table/homo-sapiens/ntrk1.html?sort=official) | YWHAH | GNAI1 |  | EIF4E1B |  | FYN |  |
|  | [GAS6](https://thebiogrid.org/108891/table/homo-sapiens/gas6.html?sort=official) | [TMEM68](https://thebiogrid.org/126482/table/homo-sapiens/tmem68.html?sort=official) | HSF4 | RIMBP3 | [DYNLL1](https://thebiogrid.org/114206/table/homo-sapiens/dynll1.html?sort=official) | [OCLN](https://thebiogrid.org/111004/table/homo-sapiens/ocln.html?sort=official) | YWHAQ | GNAQ |  | EIF4E2 |  | HCK |  |
|  | [GLRB](https://thebiogrid.org/109005/table/homo-sapiens/glrb.html?sort=official) | [TMEM9B](https://thebiogrid.org/121182/table/homo-sapiens/tmem9b.html?sort=official) | HSFX1 | RPS6KA4 | [DYNLL2](https://thebiogrid.org/126680/table/homo-sapiens/dynll2.html?sort=official) | [PCM1](https://thebiogrid.org/111139/table/homo-sapiens/pcm1.html?sort=official) | YWHAZ | [GOLT1B](https://thebiogrid.org/119232/table/homo-sapiens/golt1b.html?sort=official) |  | EIF4E3 |  | IFNG |  |
|  | [GP1BB](https://thebiogrid.org/109074/table/homo-sapiens/gp1bb.html?sort=official) | [TMX4](https://thebiogrid.org/121120/table/homo-sapiens/tmx4.html?sort=official) | HSFX2 | RPS6KA5 | [E2F1](https://thebiogrid.org/108201/table/homo-sapiens/e2f1.html?sort=official) | [PDE4D](https://thebiogrid.org/111170/table/homo-sapiens/pde4d.html?sort=official) | [ZBTB21](https://thebiogrid.org/119066/table/homo-sapiens/zbtb21.html?sort=official) | [GPR21](https://thebiogrid.org/109103/table/homo-sapiens/gpr21.html?sort=official) |  | EIF4EBP3 |  | [ITGA4](https://thebiogrid.org/109883/table/homo-sapiens/itga4.html?sort=official) |  |
|  | [GPR50](https://thebiogrid.org/114674/table/homo-sapiens/gpr50.html?sort=official) | [XYLT2](https://thebiogrid.org/122081/table/homo-sapiens/xylt2.html?sort=official) | HSFY1 | SPRY1 | [EGFR](https://thebiogrid.org/108276/table/homo-sapiens/egfr.html?sort=official) | PER2 | [ZDBF2](https://thebiogrid.org/121710/table/homo-sapiens/zdbf2.html?sort=official) | GRK6 |  | EIF4G1 |  | ITGAM |  |
|  | [GPR89A](https://thebiogrid.org/575849/table/homo-sapiens/gpr89a.html?sort=official) |  | HSFY2 | [SPRY2](https://thebiogrid.org/115547/table/homo-sapiens/spry2.html?sort=official) | [ELAVL1](https://thebiogrid.org/108309/table/homo-sapiens/elavl1.html?sort=official) | [PHLPP1](https://thebiogrid.org/116843/table/homo-sapiens/phlpp1.html?sort=official) | [ZNF618](https://thebiogrid.org/125407/table/homo-sapiens/znf618.html?sort=official) | [HIF1A](https://thebiogrid.org/107639/table/homo-sapiens/ccr5.html?sort=official) |  | EIF4G3 |  | ITGB2 |  |
|  | [GPRC5C](https://thebiogrid.org/120980/table/homo-sapiens/gprc5c.html?sort=official) |  | JUN | SRF | [EPM2AIP1](https://thebiogrid.org/115186/table/homo-sapiens/epm2aip1.html?sort=official) | [PIP5K1A](https://thebiogrid.org/113983/table/homo-sapiens/pip5k1a.html?sort=official) | ZNF705B | [HLA-B](https://thebiogrid.org/109351/table/homo-sapiens/hla-b.html?sort=official) |  | [EIF4H](https://thebiogrid.org/113297/table/homo-sapiens/eif4h.html?sort=official) |  | LAT |  |
|  | [GYLTL1B](https://thebiogrid.org/125666/table/homo-sapiens/gyltl1b.html?sort=official) |  | JUNB | TRAF2 | [ERF](https://thebiogrid.org/108388/table/homo-sapiens/erf.html?sort=official) | [POTEB3](https://thebiogrid.org/130821/table/homo-sapiens/poteb3.html?sort=official) | ZNF705D | [IFNLR1](https://thebiogrid.org/127873/table/homo-sapiens/ifnlr1.html?sort=official) |  | EIF5 |  | [LGALS3](https://thebiogrid.org/110149/table/homo-sapiens/lgals3.html?sort=official) |  |
|  | [HLA-C](https://thebiogrid.org/109352/table/homo-sapiens/hla-c.html?sort=official) |  | JUND | TRIM23 | [FAM110D](https://thebiogrid.org/123003/table/homo-sapiens/fam110d.html?sort=official) | [POTEC](https://thebiogrid.org/132700/table/homo-sapiens/potec.html?sort=official) | ZNF705G | IL8 |  | [FAM24B](https://thebiogrid.org/128226/table/homo-sapiens/fam24b.html?sort=official) |  | LILRA6 |  |
|  | [INTS4](https://thebiogrid.org/124909/table/homo-sapiens/ints4.html?sort=official) |  | KIFC3 | TRIP6 | FAM123B | [PPP1CC](https://thebiogrid.org/111495/table/homo-sapiens/ppp1cc.html?sort=official) |  | [IPPK](https://thebiogrid.org/122279/table/homo-sapiens/ippk.html?sort=official) |  | [FN1](https://thebiogrid.org/108621/table/homo-sapiens/fn1.html?sort=official) |  | LYN |  |
|  | ITGB7 |  | KRT15 | TSGA10 | [FAM170A](https://thebiogrid.org/130989/table/homo-sapiens/fam170a.html?sort=official) | [PPP1R14A](https://thebiogrid.org/125153/table/homo-sapiens/ppp1r14a.html?sort=official) |  | ITCH |  | HERC5 |  | MYH2 |  |
|  | [KIAA1244](https://thebiogrid.org/121457/table/homo-sapiens/kiaa1244.html?sort=official) |  | KRT40 |  | [FAM83B](https://thebiogrid.org/128805/table/homo-sapiens/fam83b.html?sort=official) | [PPP2CA](https://thebiogrid.org/111507/table/homo-sapiens/ppp2ca.html?sort=official) |  | ITIH4 |  | [HSPA5](https://thebiogrid.org/109541/table/homo-sapiens/hspa5.html?sort=official) |  | MYO1C |  |
|  | [KIAA1586](https://thebiogrid.org/121717/table/homo-sapiens/kiaa1586.html?sort=official) |  | KRTAP10-1 |  | [FAM83D](https://thebiogrid.org/123547/table/homo-sapiens/fam83d.html?sort=official) | [RASAL2](https://thebiogrid.org/114848/table/homo-sapiens/rasal2.html?sort=official) |  | JAK2 |  | [HSPB1](https://thebiogrid.org/109547/table/homo-sapiens/hspb1.html?sort=official) |  | NCKAP1 |  |
|  | KLRB1 |  | KRTAP10-11 |  | [FAM83G](https://thebiogrid.org/569917/table/homo-sapiens/fam83g.html?sort=official) | [RCC1](https://thebiogrid.org/107529/table/homo-sapiens/rcc1.html?sort=official) |  | [JAK3](https://thebiogrid.org/112288/table/homo-sapiens/cxcl12.html?sort=official) |  | ISG15 |  | [NFYC](https://thebiogrid.org/110868/table/homo-sapiens/nfyc.html?sort=official) |  |
|  | KLRF1 |  | KRTAP10-3 |  | [FAM83H](https://thebiogrid.org/130292/table/homo-sapiens/fam83h.html?sort=official) | RHOB |  | [KCNK1](https://thebiogrid.org/200907/table/mus-musculus/kcnk1.html?sort=official) |  | [ITGA4](https://thebiogrid.org/109883/table/homo-sapiens/itga4.html?sort=official) |  | PAK1 |  |
|  | [LCLAT1](https://thebiogrid.org/128972/table/homo-sapiens/lclat1.html?sort=official) |  | KRTAP10-5 |  | FOXO1 | RIOK2 |  | LCK |  | [MAGEA1](https://thebiogrid.org/110274/table/homo-sapiens/magea1.html?sort=official) |  | PIK3CA |  |
|  | [LMBR1](https://thebiogrid.org/122137/table/homo-sapiens/lmbr1.html?sort=official) |  | KRTAP10-7 |  | FRAT1 | [RPA1](https://thebiogrid.org/112037/table/homo-sapiens/rpa1.html?sort=official) |  | [LPAR1](https://thebiogrid.org/108226/table/homo-sapiens/lpar1.html?sort=official) |  | [MAGEA6](https://thebiogrid.org/110279/table/homo-sapiens/magea6.html?sort=official) |  | PIK3CB |  |
|  | [LMBRD2](https://thebiogrid.org/124923/table/homo-sapiens/lmbrd2.html?sort=official) |  | KRTAP10-8 |  | FRAT1 | [RPA2](https://thebiogrid.org/112038/table/homo-sapiens/rpa2.html?sort=official) |  | [MYBL2](https://thebiogrid.org/110690/table/homo-sapiens/mybl2.html?sort=official) |  | [MCM2](https://thebiogrid.org/110339/table/homo-sapiens/mcm2.html?sort=official) |  | PIK3R1 |  |
| Partner protein, color code: Black, results from BioGrid; Blue, results from STRING; Red, results from BioGrid and STRING. | | | | | | | | | | | | | |

*(continue on next page)*

| **Supplementary Table 3. Predicted functional partners proteins.** | | | | | | | | | | | | |
| --- | --- | --- | --- | --- | --- | --- | --- | --- | --- | --- | --- | --- |
| **GIT2** | | | **MS4A7** | **PRDM1** | | **PRDX3** | | **SDCBP** | | | | |
| ACTA1 | LAMB1 | [SMAD3](https://thebiogrid.org/110263/table/homo-sapiens/smad3.html?sort=official) | CPNE7 | AHR | SDC1 | ABRA | MAX | [ABI2](https://thebiogrid.org/115454/table/homo-sapiens/abi2.html?sort=official) | [EDARADD](https://thebiogrid.org/126096/table/homo-sapiens/edaradd.html?sort=official) | IL5 | [PNMA1](https://thebiogrid.org/114667/table/homo-sapiens/pnma1.html?sort=official) | [THG1L](https://thebiogrid.org/120311/table/homo-sapiens/thg1l.html?sort=official) |
| [ACTG1](https://thebiogrid.org/106586/table/homo-sapiens/actg1.html?sort=official) | LAMB2 | [SPOP](https://thebiogrid.org/113993/table/homo-sapiens/spop.html?sort=official) | HMGCL | ALDH1A1 | [SENP1](https://thebiogrid.org/118930/table/homo-sapiens/senp1.html?sort=official) | ACLY | [MCM2](https://thebiogrid.org/110339/table/homo-sapiens/mcm2.html?sort=official) | [AES](https://thebiogrid.org/106675/table/homo-sapiens/aes.html?sort=official) | [EEF1E1](https://thebiogrid.org/114898/table/homo-sapiens/eef1e1.html?sort=official) | IL5RA | [PNMA2](https://thebiogrid.org/115926/table/homo-sapiens/pnma2.html?sort=official) | [TIFA](https://thebiogrid.org/124962/table/homo-sapiens/tifa.html?sort=official) |
| [ACTN1](https://thebiogrid.org/106602/table/homo-sapiens/actn1.html?sort=official) | LAMB3 | SRC | MS4A1 | [ATXN1](https://thebiogrid.org/112217/table/homo-sapiens/atxn1.html?sort=official) | SPI1 | [ARMCX3](https://thebiogrid.org/119614/table/homo-sapiens/armcx3.html?sort=official) | [MSN](https://thebiogrid.org/201534/table/mus-musculus/msn.html?sort=official) | AIM1 | EFNB1 | [IL7R](https://thebiogrid.org/109789/table/homo-sapiens/il7r.html?sort=official) | [PPP6R2](https://thebiogrid.org/115053/table/homo-sapiens/ppp6r2.html?sort=official) | [TMED2](https://thebiogrid.org/207909/table/mus-musculus/tmed2.html?sort=official) |
| ADRBK1 | LAMC1 | [SYNC](https://thebiogrid.org/123502/table/homo-sapiens/sync.html?sort=official) | MS4A4A | BACH2 | SPIB | ATOX1 | MYC | [AIMP1](https://thebiogrid.org/114679/table/homo-sapiens/aimp1.html?sort=official) | EFNB2 | [INO80E](https://thebiogrid.org/129701/table/homo-sapiens/ino80e.html?sort=official) | [PRPF38A](https://thebiogrid.org/124382/table/homo-sapiens/prpf38a.html?sort=official) | [TMEM223](https://thebiogrid.org/122517/table/homo-sapiens/tmem223.html?sort=official) |
| AGRN | LAMC2 | [TAX1BP3](https://thebiogrid.org/119061/table/homo-sapiens/tax1bp3.html?sort=official) | SLC35F6 | [BMX](https://thebiogrid.org/107128/table/homo-sapiens/bmx.html?sort=official) | [SRC](https://thebiogrid.org/112592/table/homo-sapiens/src.html?sort=official) | [ATP5J](https://thebiogrid.org/107006/table/homo-sapiens/atp5j.html?sort=official) | [NEK6](https://thebiogrid.org/116000/table/homo-sapiens/nek6.html?sort=official) | [AIMP2](https://thebiogrid.org/113684/table/homo-sapiens/aimp2.html?sort=official) | [EIF1AD](https://thebiogrid.org/124012/table/homo-sapiens/eif1ad.html?sort=official) | [ITGA4](https://thebiogrid.org/109883/table/homo-sapiens/itga4.html?sort=official) | [PRPF40A](https://thebiogrid.org/120792/table/homo-sapiens/prpf40a.html?sort=official) | [TMEM239](https://thebiogrid.org/940453/table/homo-sapiens/tmem239.html?sort=official) |
| AGT | LAMC3 | [TFPT](https://thebiogrid.org/118931/table/homo-sapiens/tfpt.html?sort=official) |  | CD99 | STAT5A | [BIRC2](https://thebiogrid.org/106826/table/homo-sapiens/birc2.html?sort=official) | [NLRX1](https://thebiogrid.org/122796/table/homo-sapiens/nlrx1.html?sort=official) | [ANKRD28](https://thebiogrid.org/116847/table/homo-sapiens/ankrd28.html?sort=official) | [EIF5A2](https://thebiogrid.org/121162/table/homo-sapiens/eif5a2.html?sort=official) | [JUNB](https://thebiogrid.org/200872/table/mus-musculus/junb.html?sort=official) | [PRR13](https://thebiogrid.org/119965/table/homo-sapiens/prr13.html?sort=official) | [TNFAIP8](https://thebiogrid.org/117344/table/homo-sapiens/tnfaip8.html?sort=official) |
| ARF1 | [LMNB1](https://thebiogrid.org/110187/table/homo-sapiens/lmnb1.html?sort=official) | TGFB1I1 |  | CIITA | STAT5B | CAT | NME8 | [ANKRD40](https://thebiogrid.org/124821/table/homo-sapiens/ankrd40.html?sort=official) | [ELAVL1](https://thebiogrid.org/108309/table/homo-sapiens/elavl1.html?sort=official) | [KARS](https://thebiogrid.org/109938/table/homo-sapiens/kars.html?sort=official) | [PSMC6](https://thebiogrid.org/111679/table/homo-sapiens/psmc6.html?sort=official) | [TNFRSF10A](https://thebiogrid.org/114325/table/homo-sapiens/tnfrsf10a.html?sort=official) |
| ARF6 | [LPXN](https://thebiogrid.org/114801/table/homo-sapiens/lpxn.html?sort=official) | [THAP11](https://thebiogrid.org/121453/table/homo-sapiens/thap11.html?sort=official) |  | DKK1 | SUMO1 | [CCNB1](https://thebiogrid.org/107332/table/homo-sapiens/ccnb1.html?sort=official) | [NUSAP1](https://thebiogrid.org/119376/table/homo-sapiens/nusap1.html?sort=official) | [ANP32B](https://thebiogrid.org/115795/table/homo-sapiens/anp32b.html?sort=official) | [ENOX1](https://thebiogrid.org/120385/table/homo-sapiens/enox1.html?sort=official) | [KCNH1](https://thebiogrid.org/109958/table/homo-sapiens/kcnh1.html?sort=official) | [PSME2](https://thebiogrid.org/111693/table/homo-sapiens/psme2.html?sort=official) | TOX |
| ARHGEF6 | [MAFF](https://thebiogrid.org/117264/table/homo-sapiens/maff.html?sort=official) | [TMEM154](https://thebiogrid.org/128404/table/homo-sapiens/tmem154.html?sort=official) |  | [DLG1](https://thebiogrid.org/108083/table/homo-sapiens/dlg1.html?sort=official) | TBX21 | [CD2AP](https://thebiogrid.org/198584/table/mus-musculus/cd2ap.html?sort=official) | [OBSL1](https://thebiogrid.org/116944/table/homo-sapiens/obsl1.html?sort=official) | [APIP](https://thebiogrid.org/119265/table/homo-sapiens/apip.html?sort=official) | EPHB2 | [KCTD1](https://thebiogrid.org/129804/table/homo-sapiens/kctd1.html?sort=official) | [PSTPIP1](https://thebiogrid.org/114513/table/homo-sapiens/pstpip1.html?sort=official) | TP53 |
| ARHGEF7 | [MAX](https://thebiogrid.org/110319/table/homo-sapiens/max.html?sort=official) | [TMOD4](https://thebiogrid.org/118898/table/homo-sapiens/tmod4.html?sort=official) |  | [DPYSL3](https://thebiogrid.org/108143/table/homo-sapiens/dpysl3.html?sort=official) | TLE1 | [CDC26](https://thebiogrid.org/211475/table/mus-musculus/cdc26.html?sort=official) | [OPA1](https://thebiogrid.org/111024/table/homo-sapiens/opa1.html?sort=official) | [BCL2L15](https://thebiogrid.org/136727/table/homo-sapiens/bcl2l15.html?sort=official) | [EPRS](https://thebiogrid.org/108372/table/homo-sapiens/eprs.html?sort=official) | [KCTD6](https://thebiogrid.org/128350/table/homo-sapiens/kctd6.html?sort=official) | [PTEN](https://thebiogrid.org/111700/table/homo-sapiens/pten.html?sort=official) | TPH2 |
| [ARNTL](https://thebiogrid.org/106899/table/homo-sapiens/arntl.html?sort=official) | [MED4](https://thebiogrid.org/118849/table/homo-sapiens/med4.html?sort=official) | [TNFAIP3](https://thebiogrid.org/112983/table/homo-sapiens/tnfaip3.html?sort=official) |  | EHMT2 | [TLE2](https://thebiogrid.org/112944/table/homo-sapiens/tle2.html?sort=official) | [CDC42](https://thebiogrid.org/107433/table/homo-sapiens/cdc42.html?sort=official) | P4HB | [C11orf57](https://thebiogrid.org/120512/table/homo-sapiens/c11orf57.html?sort=official) | [ERICH2](https://thebiogrid.org/130024/table/homo-sapiens/erich2.html?sort=official) | [KCTD9](https://thebiogrid.org/120153/table/homo-sapiens/kctd9.html?sort=official) | PTPRJ | [TRAF5](https://thebiogrid.org/113040/table/homo-sapiens/traf5.html?sort=official) |
| ASS1 | [MOV10](https://thebiogrid.org/110484/table/homo-sapiens/mov10.html?sort=official) | [TNIP1](https://thebiogrid.org/115602/table/homo-sapiens/tnip1.html?sort=official) |  | [ELAVL1](https://thebiogrid.org/108309/table/homo-sapiens/elavl1.html?sort=official) | TLR2 | [CDK2](https://thebiogrid.org/107452/table/homo-sapiens/cdk2.html?sort=official) | [PARD6A](https://thebiogrid.org/119157/table/homo-sapiens/pard6a.html?sort=official) | [C11orf68](https://thebiogrid.org/123702/table/homo-sapiens/c11orf68.html?sort=official) | [EWSR1](https://thebiogrid.org/108431/table/homo-sapiens/ewsr1.html?sort=official) | [KHDRBS2](https://thebiogrid.org/128436/table/homo-sapiens/khdrbs2.html?sort=official) | [PTS](https://thebiogrid.org/111769/table/homo-sapiens/pts.html?sort=official) | [TRAF6](https://thebiogrid.org/115454/table/homo-sapiens/abi2.html?sort=official) |
| [ATF5](https://thebiogrid.org/116487/table/homo-sapiens/atf5.html?sort=official) | [MVP](https://thebiogrid.org/115286/table/homo-sapiens/mvp.html?sort=official) | [TRAF1](https://thebiogrid.org/113037/table/homo-sapiens/traf1.html?sort=official) |  | ELL2 | TOP3B | [COPS5](https://thebiogrid.org/116183/table/homo-sapiens/cops5.html?sort=official) | PARK7 | [C16orf58](https://thebiogrid.org/122269/table/homo-sapiens/c16orf58.html?sort=official) | [EZH2](https://thebiogrid.org/108446/table/homo-sapiens/ezh2.html?sort=official) | [KLHL12](https://thebiogrid.org/121890/table/homo-sapiens/klhl12.html?sort=official) | [PUF60](https://thebiogrid.org/116502/table/homo-sapiens/puf60.html?sort=official) | [TRIM27](https://thebiogrid.org/111919/table/homo-sapiens/trim27.html?sort=official) |
| [C4BPA](https://thebiogrid.org/107183/table/homo-sapiens/c4bpa.html?sort=official) | NBN | [TSN](https://thebiogrid.org/113098/table/homo-sapiens/tsn.html?sort=official) |  | ETS1 | TP53 | [CUL1](https://thebiogrid.org/114032/table/homo-sapiens/cul1.html?sort=official) | [PCNA](https://thebiogrid.org/111142/table/homo-sapiens/pcna.html?sort=official) | [C1orf109](https://thebiogrid.org/120293/table/homo-sapiens/c1orf109.html?sort=official) | [FAM118A](https://thebiogrid.org/120339/table/homo-sapiens/fam118a.html?sort=official) | [KLHL2](https://thebiogrid.org/116431/table/homo-sapiens/klhl2.html?sort=official) | [PYCRL](https://thebiogrid.org/122418/table/homo-sapiens/pycrl.html?sort=official) | [TRIM32](https://thebiogrid.org/116608/table/homo-sapiens/trim32.html?sort=official) |
| [CALCOCO2](https://thebiogrid.org/115535/table/homo-sapiens/calcoco2.html?sort=official) | NCK1 | [TSSC1](https://thebiogrid.org/113111/table/homo-sapiens/tssc1.html?sort=official) |  | FOS | [UBE4A](https://thebiogrid.org/114757/table/homo-sapiens/ube4a.html?sort=official) | [CUL2](https://thebiogrid.org/114031/table/homo-sapiens/cul2.html?sort=official) | PDIA2 | CA8 | [FAM118B](https://thebiogrid.org/122742/table/homo-sapiens/fam118b.html?sort=official) | [KRTAP10-3](https://thebiogrid.org/132133/table/homo-sapiens/krtap10-3.html?sort=official) | [QARS](https://thebiogrid.org/111797/table/homo-sapiens/qars.html?sort=official) | [TRIM38](https://thebiogrid.org/115738/table/homo-sapiens/trim38.html?sort=official) |
| [CAMK4](https://thebiogrid.org/107264/table/homo-sapiens/camk4.html?sort=official) | NCK2 | [TUFT1](https://thebiogrid.org/113137/table/homo-sapiens/tuft1.html?sort=official) |  | [GTF2I](https://thebiogrid.org/109224/table/homo-sapiens/gtf2i.html?sort=official) | VSX2 | [CUL3](https://thebiogrid.org/114030/table/homo-sapiens/cul3.html?sort=official) | PDIA3 | CADM1 | [FAM8A1](https://thebiogrid.org/119541/table/homo-sapiens/fam8a1.html?sort=official) | [KRTAP10-7](https://thebiogrid.org/132126/table/homo-sapiens/krtap10-7.html?sort=official) | RAB5A | [TRIM54](https://thebiogrid.org/121415/table/homo-sapiens/trim54.html?sort=official) |
| [CCDC132](https://thebiogrid.org/120750/table/homo-sapiens/ccdc132.html?sort=official) | [NCKAP5L](https://thebiogrid.org/121726/table/homo-sapiens/nckap5l.html?sort=official) | [UBQLN1](https://thebiogrid.org/119007/table/homo-sapiens/ubqln1.html?sort=official) |  | GZMB | XBP1 | [CUL4B](https://thebiogrid.org/114028/table/homo-sapiens/cul4b.html?sort=official) | [PFKL](https://thebiogrid.org/111232/table/homo-sapiens/pfkl.html?sort=official) | [CADPS](https://thebiogrid.org/114174/table/homo-sapiens/cadps.html?sort=official) | [FAM9B](https://thebiogrid.org/128131/table/homo-sapiens/fam9b.html?sort=official) | [KRTAP5-9](https://thebiogrid.org/110044/table/homo-sapiens/krtap5-9.html?sort=official) | [RARS](https://thebiogrid.org/111852/table/homo-sapiens/rars.html?sort=official) | [TSPAN3](https://thebiogrid.org/115406/table/homo-sapiens/tspan3.html?sort=official) |
| [CCDC93](https://thebiogrid.org/120013/table/homo-sapiens/ccdc93.html?sort=official) | [NCKIPSD](https://thebiogrid.org/119583/table/homo-sapiens/nckipsd.html?sort=official) | [USHBP1](https://thebiogrid.org/123791/table/homo-sapiens/ushbp1.html?sort=official) |  | HDAC1 | [YES1](https://thebiogrid.org/113357/table/homo-sapiens/yes1.html?sort=official) | [CUL5](https://thebiogrid.org/113743/table/homo-sapiens/cul5.html?sort=official) | [POU5F1](https://thebiogrid.org/111456/table/homo-sapiens/pou5f1.html?sort=official) | [CALCOCO2](https://thebiogrid.org/115535/table/homo-sapiens/calcoco2.html?sort=official) | [FLAD1](https://thebiogrid.org/123221/table/homo-sapiens/flad1.html?sort=official) | [LARS](https://thebiogrid.org/119584/table/homo-sapiens/lars.html?sort=official) | [RBM39](https://thebiogrid.org/114952/table/homo-sapiens/rbm39.html?sort=official) | [UBC](https://thebiogrid.org/115795/table/homo-sapiens/anp32b.html?sort=official) |
| [CCHCR1](https://thebiogrid.org/120022/table/homo-sapiens/cchcr1.html?sort=official) | [NFKBIB](https://thebiogrid.org/110860/table/homo-sapiens/nfkbib.html?sort=official) | UTRN |  | HDAC2 | [ZBTB32](https://thebiogrid.org/117964/table/homo-sapiens/zbtb32.html?sort=official) | [CUL7](https://thebiogrid.org/115159/table/homo-sapiens/cul7.html?sort=official) | [PRDX1](https://thebiogrid.org/111089/table/homo-sapiens/prdx1.html?sort=official) | [CBR3](https://thebiogrid.org/107320/table/homo-sapiens/cbr3.html?sort=official) | [FOXP2](https://thebiogrid.org/125073/table/homo-sapiens/foxp2.html?sort=official) | [LDOC1](https://thebiogrid.org/117169/table/homo-sapiens/ldoc1.html?sort=official) | [REEP6](https://thebiogrid.org/124983/table/homo-sapiens/reep6.html?sort=official) | [UBE2A](https://thebiogrid.org/113167/table/homo-sapiens/ube2a.html?sort=official) |
| CDC42 | [NME2](https://thebiogrid.org/110895/table/homo-sapiens/nme2.html?sort=official) | [VPS51](https://thebiogrid.org/107197/table/homo-sapiens/vps51.html?sort=official) |  | [HIST1H1A](https://thebiogrid.org/109275/table/homo-sapiens/hist1h1a.html?sort=official) |  | [DCUN1D1](https://thebiogrid.org/119920/table/homo-sapiens/dcun1d1.html?sort=official) | [PRDX2](https://thebiogrid.org/112860/table/homo-sapiens/prdx2.html?sort=official) | [CCDC102B](https://thebiogrid.org/122930/table/homo-sapiens/ccdc102b.html?sort=official) | [FTH1](https://thebiogrid.org/108773/table/homo-sapiens/fth1.html?sort=official) | [LGALS2](https://thebiogrid.org/110148/table/homo-sapiens/lgals2.html?sort=official) | [REL](https://thebiogrid.org/111898/table/homo-sapiens/rel.html?sort=official) | [ULK1](https://thebiogrid.org/120512/table/homo-sapiens/c11orf57.html?sort=official) |
| CHRM1 | [NUF2](https://thebiogrid.org/123673/table/homo-sapiens/nuf2.html?sort=official) | [VPS53](https://thebiogrid.org/120563/table/homo-sapiens/vps53.html?sort=official) |  | [HIST2H3A](https://thebiogrid.org/130616/table/homo-sapiens/hist2h3a.html?sort=official) |  | [DHRS4](https://thebiogrid.org/116107/table/homo-sapiens/dhrs4.html?sort=official) | [PRDX3](https://thebiogrid.org/116136/table/homo-sapiens/prdx3.html?sort=official) | [CCDC36](https://thebiogrid.org/130947/table/homo-sapiens/ccdc36.html?sort=official) | [FTL](https://thebiogrid.org/108789/table/homo-sapiens/ftl.html?sort=official) | [LGALS8](https://thebiogrid.org/110155/table/homo-sapiens/lgals8.html?sort=official) | [RIC8A](https://thebiogrid.org/121946/table/homo-sapiens/ric8a.html?sort=official) | [ULK2](https://thebiogrid.org/123702/table/homo-sapiens/c11orf68.html?sort=official) |
| CHRNA1 | [OIP5](https://thebiogrid.org/116467/table/homo-sapiens/oip5.html?sort=official) | [YEATS4](https://thebiogrid.org/113761/table/homo-sapiens/yeats4.html?sort=official) |  | [HSP90AA1](https://thebiogrid.org/109552/table/homo-sapiens/hsp90aa1.html?sort=official) |  | DNAJC10 | [PRDX4](https://thebiogrid.org/115800/table/homo-sapiens/prdx4.html?sort=official) | [CCDC53](https://thebiogrid.org/119225/table/homo-sapiens/ccdc53.html?sort=official) | [FYN](https://thebiogrid.org/108810/table/homo-sapiens/fyn.html?sort=official) | [LITAF](https://thebiogrid.org/114893/table/homo-sapiens/litaf.html?sort=official) | [RNF11](https://thebiogrid.org/117941/table/homo-sapiens/rnf11.html?sort=official) | [UNK](https://thebiogrid.org/124536/table/homo-sapiens/unk.html?sort=official) |
| [CLOCK](https://thebiogrid.org/114944/table/homo-sapiens/clock.html?sort=official) | PAK1 | YES1 |  | [HSPA5](https://thebiogrid.org/109541/table/homo-sapiens/hspa5.html?sort=official) |  | [DUSP13](https://thebiogrid.org/119380/table/homo-sapiens/dusp13.html?sort=official) | PRDX5 | CD6 | FZD1 | [LMNA](https://thebiogrid.org/110186/table/homo-sapiens/lmna.html?sort=official) | [RNH1](https://thebiogrid.org/111977/table/homo-sapiens/rnh1.html?sort=official) | [VCAM1](https://thebiogrid.org/113255/table/homo-sapiens/vcam1.html?sort=official) |
| [CORO1A](https://thebiogrid.org/116322/table/homo-sapiens/coro1a.html?sort=official) | PAK2 |  |  | [HSPA9](https://thebiogrid.org/109545/table/homo-sapiens/hspa9.html?sort=official) |  | FANCA | [PSMD6](https://thebiogrid.org/211457/table/mus-musculus/psmd6.html?sort=official) | CD63 | FZD2 | [LSM6](https://thebiogrid.org/116328/table/homo-sapiens/lsm6.html?sort=official) | [ROPN1](https://thebiogrid.org/120138/table/homo-sapiens/ropn1.html?sort=official) | [WASL](https://thebiogrid.org/114466/table/homo-sapiens/wasl.html?sort=official) |
| CTTN | PAK3 |  |  | [HSPD1](https://thebiogrid.org/109561/table/homo-sapiens/hspd1.html?sort=official) |  | FANCC | PSME1 | [CD83](https://thebiogrid.org/114721/table/homo-sapiens/cd83.html?sort=official) | FZD3 | [LTBR](https://thebiogrid.org/110233/table/homo-sapiens/ltbr.html?sort=official) | [RP9P](https://thebiogrid.org/137267/table/homo-sapiens/rp9p.html?sort=official) | [WRAP73](https://thebiogrid.org/119068/table/homo-sapiens/wrap73.html?sort=official) |
| DAG1 | PAK4 |  |  | IDO1 |  | FANCG | [PTP4A2](https://thebiogrid.org/113747/table/homo-sapiens/ptp4a2.html?sort=official) | [CDA](https://thebiogrid.org/107416/table/homo-sapiens/cda.html?sort=official) | FZD7 | [LZTFL1](https://thebiogrid.org/120062/table/homo-sapiens/lztfl1.html?sort=official) | [RPL28](https://thebiogrid.org/112077/table/homo-sapiens/rpl28.html?sort=official) | [ZBTB14](https://thebiogrid.org/113373/table/homo-sapiens/zbtb14.html?sort=official) |
| DMD | PAK6 |  |  | IFNA1 |  | [FN1](https://thebiogrid.org/108621/table/homo-sapiens/fn1.html?sort=official) | RNASET2 | [CDC34](https://thebiogrid.org/107432/table/homo-sapiens/cdc34.html?sort=official) | FZD8 | [MAD2L1](https://thebiogrid.org/110260/table/homo-sapiens/mad2l1.html?sort=official) | [RTN1](https://thebiogrid.org/112165/table/homo-sapiens/rtn1.html?sort=official) | [ZBTB8A](https://thebiogrid.org/575543/table/homo-sapiens/zbtb8a.html?sort=official) |
| [DZIP3](https://thebiogrid.org/115021/table/homo-sapiens/dzip3.html?sort=official) | PAK7 |  |  | [IKZF3](https://thebiogrid.org/116484/table/homo-sapiens/ikzf3.html?sort=official) |  | FOXO3 | [RPS4Y1](https://thebiogrid.org/112106/table/homo-sapiens/rps4y1.html?sort=official) | [CDCP1](https://thebiogrid.org/122336/table/homo-sapiens/cdcp1.html?sort=official) | [GAG](https://thebiogrid.org/1205537/table/human-immunodeficiency-virus-1/gag.html?sort=official) | [MANSC1](https://thebiogrid.org/120101/table/homo-sapiens/mansc1.html?sort=official) | [RUNDC3A](https://thebiogrid.org/116106/table/homo-sapiens/rundc3a.html?sort=official) | [ZCCHC10](https://thebiogrid.org/120174/table/homo-sapiens/zcchc10.html?sort=official) |
| [E2F2](https://thebiogrid.org/108202/table/homo-sapiens/e2f2.html?sort=official) | [PCLO](https://thebiogrid.org/118178/table/homo-sapiens/pclo.html?sort=official) |  |  | IL21 |  | GAPDH | RPS6KC1 | CDH1 | GNG10 | [MAPRE3](https://thebiogrid.org/116584/table/homo-sapiens/mapre3.html?sort=official) | [SAMD1](https://thebiogrid.org/124704/table/homo-sapiens/samd1.html?sort=official) | [ZCCHC17](https://thebiogrid.org/119598/table/homo-sapiens/zcchc17.html?sort=official) |
| [EDC4](https://thebiogrid.org/117171/table/homo-sapiens/edc4.html?sort=official) | [PHF21A](https://thebiogrid.org/119468/table/homo-sapiens/phf21a.html?sort=official) |  |  | IL29 |  | GLRX | [SAMM50](https://thebiogrid.org/212974/table/mus-musculus/samm50.html?sort=official) | CDH3 | [GNMT](https://thebiogrid.org/118081/table/homo-sapiens/gnmt.html?sort=official) | [MARS](https://thebiogrid.org/110311/table/homo-sapiens/mars.html?sort=official) | [SCLT1](https://thebiogrid.org/126318/table/homo-sapiens/sclt1.html?sort=official) |  |
| [EXOC1](https://thebiogrid.org/120881/table/homo-sapiens/exoc1.html?sort=official) | [PHLDB3](https://thebiogrid.org/575902/table/homo-sapiens/phldb3.html?sort=official) |  |  | IL5 |  | GLRX2 | [SMC6](https://thebiogrid.org/122802/table/homo-sapiens/smc6.html?sort=official) | [CDIPT](https://thebiogrid.org/115692/table/homo-sapiens/cdipt.html?sort=official) | [GPATCH11](https://thebiogrid.org/128977/table/homo-sapiens/gpatch11.html?sort=official) | [MBD3](https://thebiogrid.org/119788/table/homo-sapiens/mbd3.html?sort=official) | SDC1 |  |
| [FBXO28](https://thebiogrid.org/116826/table/homo-sapiens/fbxo28.html?sort=official) | [PKN3](https://thebiogrid.org/118978/table/homo-sapiens/pkn3.html?sort=official) |  |  | IRF1 |  | [GORASP2](https://thebiogrid.org/117479/table/homo-sapiens/gorasp2.html?sort=official) | [SNCA](https://thebiogrid.org/112506/table/homo-sapiens/snca.html?sort=official) | CDK2AP1 | GPR37 | [MED4](https://thebiogrid.org/118849/table/homo-sapiens/med4.html?sort=official) | SDC2 |  |
| FYN | [POLR1B](https://thebiogrid.org/123926/table/homo-sapiens/polr1b.html?sort=official) |  |  | IRF2 |  | GPX1 | SOD2 | [CEP170](https://thebiogrid.org/115193/table/homo-sapiens/cep170.html?sort=official) | [GPRC5B](https://thebiogrid.org/119688/table/homo-sapiens/gprc5b.html?sort=official) | [MID2](https://thebiogrid.org/116231/table/homo-sapiens/mid2.html?sort=official) | SDC4 |  |
| [GCH1](https://thebiogrid.org/108913/table/homo-sapiens/gch1.html?sort=official) | [PROX1](https://thebiogrid.org/111613/table/homo-sapiens/prox1.html?sort=official) |  |  | IRF4 |  | GPX2 | SOD3 | [CEP55](https://thebiogrid.org/120465/table/homo-sapiens/cep55.html?sort=official) | GRIA1 | MMP11 | SDCBP2 |  |
| GIT1 | [PSMD9](https://thebiogrid.org/111687/table/homo-sapiens/psmd9.html?sort=official) |  |  | IRF5 |  | GPX3 | SPEN | [CGGBP1](https://thebiogrid.org/114115/table/homo-sapiens/cggbp1.html?sort=official) | [GRIA2](https://thebiogrid.org/109148/table/homo-sapiens/gria2.html?sort=official) | [MRAP2](https://thebiogrid.org/125195/table/homo-sapiens/mrap2.html?sort=official) | [SDCCAG3](https://thebiogrid.org/116021/table/homo-sapiens/sdccag3.html?sort=official) |  |
| [GIT2](https://thebiogrid.org/115154/table/homo-sapiens/git2.html?sort=official) | PTK2 |  |  | JUN |  | GPX4 | [SPRED2](https://thebiogrid.org/227830/table/mus-musculus/spred2.html?sort=official) | [CHMP4B](https://thebiogrid.org/217613/table/mus-musculus/chmp4b.html?sort=official) | [GRIA3](https://thebiogrid.org/109149/table/homo-sapiens/gria3.html?sort=official) | [MRFAP1L1](https://thebiogrid.org/125400/table/homo-sapiens/mrfap1l1.html?sort=official) | [SET](https://thebiogrid.org/112316/table/homo-sapiens/set.html?sort=official) |  |
| [GOLGA5](https://thebiogrid.org/115275/table/homo-sapiens/golga5.html?sort=official) | [PTRF](https://thebiogrid.org/129767/table/homo-sapiens/ptrf.html?sort=official) |  |  | KDM1A |  | GPX5 | SRXN1 | [CLK2](https://thebiogrid.org/107607/table/homo-sapiens/clk2.html?sort=official) | [GRIA4](https://thebiogrid.org/109150/table/homo-sapiens/gria4.html?sort=official) | [MTUS2](https://thebiogrid.org/116880/table/homo-sapiens/mtus2.html?sort=official) | [SIAH1](https://thebiogrid.org/112372/table/homo-sapiens/siah1.html?sort=official) |  |
| [GRIPAP1](https://thebiogrid.org/121210/table/homo-sapiens/gripap1.html?sort=official) | PXN |  |  | LGALS3 |  | GPX6 | [TERF2](https://thebiogrid.org/112873/table/homo-sapiens/terf2.html?sort=official) | [CLK3](https://thebiogrid.org/107609/table/homo-sapiens/clk3.html?sort=official) | GRIK1 | [NADK](https://thebiogrid.org/122408/table/homo-sapiens/nadk.html?sort=official) | SLC6A5 |  |
| [GUSB](https://thebiogrid.org/109245/table/homo-sapiens/gusb.html?sort=official) | [QPRT](https://thebiogrid.org/117035/table/homo-sapiens/qprt.html?sort=official) |  |  | MIF |  | GPX7 | [TNFRSF1A](https://thebiogrid.org/112986/table/homo-sapiens/tnfrsf1a.html?sort=official) | [CMTM5](https://thebiogrid.org/125486/table/homo-sapiens/cmtm5.html?sort=official) | GRIK2 | [NAGK](https://thebiogrid.org/120728/table/homo-sapiens/nagk.html?sort=official) | [SMARCA2](https://thebiogrid.org/112479/table/homo-sapiens/smarca2.html?sort=official) |  |
| [HAUS6](https://thebiogrid.org/120160/table/homo-sapiens/haus6.html?sort=official) | [RABEP1](https://thebiogrid.org/114583/table/homo-sapiens/rabep1.html?sort=official) |  |  | [MS4A1](https://thebiogrid.org/107369/table/homo-sapiens/ms4a1.html?sort=official) |  | GPX8 | TXN | [CNOT1](https://thebiogrid.org/116660/table/homo-sapiens/cnot1.html?sort=official) | [GRM2](https://thebiogrid.org/109169/table/homo-sapiens/grm2.html?sort=official) | [NECAB2](https://thebiogrid.org/120035/table/homo-sapiens/necab2.html?sort=official) | SNTA1 |  |
| [HGD](https://thebiogrid.org/109329/table/homo-sapiens/hgd.html?sort=official) | [RABGAP1L](https://thebiogrid.org/115239/table/homo-sapiens/rabgap1l.html?sort=official) |  |  | MTA3 |  | GSR | TXN2 | [COX4I1](https://thebiogrid.org/107720/table/homo-sapiens/cox4i1.html?sort=official) | [GRM3](https://thebiogrid.org/109170/table/homo-sapiens/grm3.html?sort=official) | NF2 | SOX4 |  |
| [HMG20A](https://thebiogrid.org/115643/table/homo-sapiens/hmg20a.html?sort=official) | [RABGEF1](https://thebiogrid.org/118154/table/homo-sapiens/rabgef1.html?sort=official) |  |  | MYC |  | [GTF3C4](https://thebiogrid.org/234628/table/mus-musculus/gtf3c4.html?sort=official) | TXNDC2 | [CRYAA](https://thebiogrid.org/107799/table/homo-sapiens/cryaa.html?sort=official) | [GRM7](https://thebiogrid.org/109174/table/homo-sapiens/grm7.html?sort=official) | NFASC | SRC |  |
| [HNRNPUL1](https://thebiogrid.org/116281/table/homo-sapiens/hnrnpul1.html?sort=official) | [RABGGTB](https://thebiogrid.org/111814/table/homo-sapiens/rabggtb.html?sort=official) |  |  | MYH6 |  | [H2AFX](https://thebiogrid.org/109268/table/homo-sapiens/h2afx.html?sort=official) | TXNDC8 | CSPG4 | [HIST1H2BG](https://thebiogrid.org/113935/table/homo-sapiens/hist1h2bg.html?sort=official) | [NOTCH2NL](https://thebiogrid.org/132802/table/homo-sapiens/notch2nl.html?sort=official) | [SREK1IP1](https://thebiogrid.org/130175/table/homo-sapiens/srek1ip1.html?sort=official) |  |
| [ICAM1](https://thebiogrid.org/109610/table/homo-sapiens/icam1.html?sort=official) | RAC1 |  |  | NLRP12 |  | [HDAC5](https://thebiogrid.org/115331/table/homo-sapiens/hdac5.html?sort=official) | TXNRD1 | [CT45A3](https://thebiogrid.org/137546/table/homo-sapiens/ct45a3.html?sort=official) | [HIVEP1](https://thebiogrid.org/109343/table/homo-sapiens/hivep1.html?sort=official) | [NT5C2](https://thebiogrid.org/116627/table/homo-sapiens/nt5c2.html?sort=official) | [SRSF11](https://thebiogrid.org/114709/table/homo-sapiens/srsf11.html?sort=official) |  |
| [IKBKG](https://thebiogrid.org/114089/table/homo-sapiens/ikbkg.html?sort=official) | RAPSN |  |  | OTX2 |  | HFM1 | TXNRD2 | [CT45A5](https://thebiogrid.org/137548/table/homo-sapiens/ct45a5.html?sort=official) | [HMGB3](https://thebiogrid.org/109392/table/homo-sapiens/hmgb3.html?sort=official) | [OSTF1](https://thebiogrid.org/117747/table/homo-sapiens/ostf1.html?sort=official) | [SRSF7](https://thebiogrid.org/112330/table/homo-sapiens/srsf7.html?sort=official) |  |
| ILK | [RCOR1](https://thebiogrid.org/116796/table/homo-sapiens/rcor1.html?sort=official) |  |  | PAX2 |  | [HNRNPD](https://thebiogrid.org/109425/table/homo-sapiens/hnrnpd.html?sort=official) | TXNRD3 | [CUTC](https://thebiogrid.org/119267/table/homo-sapiens/cutc.html?sort=official) | [HNRNPC](https://thebiogrid.org/109424/table/homo-sapiens/hnrnpc.html?sort=official) | [PCDHB11](https://thebiogrid.org/121065/table/homo-sapiens/pcdhb11.html?sort=official) | [SSNA1](https://thebiogrid.org/114189/table/homo-sapiens/ssna1.html?sort=official) |  |
| ITGA1 | [RCVRN](https://thebiogrid.org/111890/table/homo-sapiens/rcvrn.html?sort=official) |  |  | PAX5 |  | IKBKB | [UBA5](https://thebiogrid.org/122964/table/homo-sapiens/uba5.html?sort=official) | CYP7B1 | [HOMEZ](https://thebiogrid.org/121645/table/homo-sapiens/homez.html?sort=official) | [PCDHGA7](https://thebiogrid.org/121048/table/homo-sapiens/pcdhga7.html?sort=official) | ST5 |  |
| ITGB1 | [RPRD1A](https://thebiogrid.org/120494/table/homo-sapiens/rprd1a.html?sort=official) |  |  | [PIAS1](https://thebiogrid.org/114124/table/homo-sapiens/pias1.html?sort=official) |  | [ITGA4](https://thebiogrid.org/109883/table/homo-sapiens/itga4.html?sort=official) | UBC | [DAK](https://thebiogrid.org/117481/table/homo-sapiens/dak.html?sort=official) | [HOXA1](https://thebiogrid.org/109438/table/homo-sapiens/hoxa1.html?sort=official) | [PCDHGB1](https://thebiogrid.org/121044/table/homo-sapiens/pcdhgb1.html?sort=official) | STX1A |  |
| [KCTD5](https://thebiogrid.org/119958/table/homo-sapiens/kctd5.html?sort=official) | [RUFY1](https://thebiogrid.org/123193/table/homo-sapiens/rufy1.html?sort=official) |  |  | POU2AF1 |  | [LNPEP](https://thebiogrid.org/110196/table/homo-sapiens/lnpep.html?sort=official) | UBQLN4 | [DARS](https://thebiogrid.org/107984/table/homo-sapiens/dars.html?sort=official) | [HPRT1](https://thebiogrid.org/109488/table/homo-sapiens/hprt1.html?sort=official) | [PCDHGB4](https://thebiogrid.org/114193/table/homo-sapiens/pcdhgb4.html?sort=official) | [SULT1B1](https://thebiogrid.org/118108/table/homo-sapiens/sult1b1.html?sort=official) |  |
| [KLHL2](https://thebiogrid.org/116431/table/homo-sapiens/klhl2.html?sort=official) | [RUSC2](https://thebiogrid.org/115187/table/homo-sapiens/rusc2.html?sort=official) |  |  | [PRDM1](https://thebiogrid.org/107108/table/homo-sapiens/prdm1.html?sort=official) |  | LRRK2 | [UCHL5](https://thebiogrid.org/119509/table/homo-sapiens/uchl5.html?sort=official) | [DCTD](https://thebiogrid.org/108003/table/homo-sapiens/dctd.html?sort=official) | [HSBP1](https://thebiogrid.org/109515/table/homo-sapiens/hsbp1.html?sort=official) | [PDCD6IP](https://thebiogrid.org/115332/table/homo-sapiens/pdcd6ip.html?sort=official) | [TARSL2](https://thebiogrid.org/125822/table/homo-sapiens/tarsl2.html?sort=official) |  |
| [KRT18](https://thebiogrid.org/110073/table/homo-sapiens/krt18.html?sort=official) | [SAFB2](https://thebiogrid.org/115022/table/homo-sapiens/safb2.html?sort=official) |  |  | PRMT5 |  | [MAGEA11](https://thebiogrid.org/110284/table/homo-sapiens/magea11.html?sort=official) | [UNK](https://thebiogrid.org/124536/table/homo-sapiens/unk.html?sort=official) | [DCTPP1](https://thebiogrid.org/122527/table/homo-sapiens/dctpp1.html?sort=official) | [IARS](https://thebiogrid.org/109605/table/homo-sapiens/iars.html?sort=official) | [PDE4DIP](https://thebiogrid.org/115017/table/homo-sapiens/pde4dip.html?sort=official) | [TDO2](https://thebiogrid.org/112858/table/homo-sapiens/tdo2.html?sort=official) |  |
| LAMA2 | [SH3GLB2](https://thebiogrid.org/121234/table/homo-sapiens/sh3glb2.html?sort=official) |  |  | PRMT7 |  | [MAP3K13](https://thebiogrid.org/114614/table/homo-sapiens/map3k13.html?sort=official) | UROD | [DMC1](https://thebiogrid.org/116316/table/homo-sapiens/dmc1.html?sort=official) | [IFI16](https://thebiogrid.org/109654/table/homo-sapiens/ifi16.html?sort=official) | PENK | [TEKT1](https://thebiogrid.org/123716/table/homo-sapiens/tekt1.html?sort=official) |  |
| LAMA3 | [SKP1](https://thebiogrid.org/112391/table/homo-sapiens/skp1.html?sort=official) |  |  | RBPJ |  | MAPK3 | [VCAM1](https://thebiogrid.org/113255/table/homo-sapiens/vcam1.html?sort=official) | [DTNBP1](https://thebiogrid.org/123857/table/homo-sapiens/dtnbp1.html?sort=official) | [IFNGR1](https://thebiogrid.org/109681/table/homo-sapiens/ifngr1.html?sort=official) | [PHC2](https://thebiogrid.org/108234/table/homo-sapiens/phc2.html?sort=official) | [TFCP2](https://thebiogrid.org/112882/table/homo-sapiens/tfcp2.html?sort=official) |  |
| LAMA5 | [SLC9A3R2](https://thebiogrid.org/114754/table/homo-sapiens/slc9a3r2.html?sort=official) |  |  | [RRAS](https://thebiogrid.org/112151/table/homo-sapiens/rras.html?sort=official) |  | [MARK2](https://thebiogrid.org/108326/table/homo-sapiens/mark2.html?sort=official) | [XIAP](https://thebiogrid.org/106828/table/homo-sapiens/xiap.html?sort=official) | [EAF1](https://thebiogrid.org/124514/table/homo-sapiens/eaf1.html?sort=official) | [IKZF1](https://thebiogrid.org/115604/table/homo-sapiens/ikzf1.html?sort=official) | PICK1 | TGFA |  |
| Partner protein, color code: Black, results from BioGrid; Blue, results from STRING; Red, results from BioGrid and STRING. | | | | | | | | | | | | |

*(continue on next page)*

| **Supplementary Table 3. Predicted functional partners proteins.** | | | | | | | |
| --- | --- | --- | --- | --- | --- | --- | --- |
| **SPG21** | | **STRN3** | | **TROVE2** | | **VNN2** | |
| [AGTRAP](https://thebiogrid.org/121356/table/homo-sapiens/agtrap.html?sort=official) | SGK494 | [ACOX2](https://thebiogrid.org/113906/table/homo-sapiens/acox2.html?sort=official) | [PBK](https://thebiogrid.org/120971/table/homo-sapiens/pbk.html?sort=official) | ACE | SNRNP70 | [ACADVL](https://thebiogrid.org/106555/table/homo-sapiens/acadvl.html?sort=official) | RHBDD2 |
| [AKIRIN2](https://thebiogrid.org/120430/table/homo-sapiens/akirin2.html?sort=official) | SPAST | APC | [PCOLCE2](https://thebiogrid.org/117746/table/homo-sapiens/pcolce2.html?sort=official) | ADPRHL2 | SSB | [ALG8](https://thebiogrid.org/122511/table/homo-sapiens/alg8.html?sort=official) | [S1PR5](https://thebiogrid.org/119793/table/homo-sapiens/s1pr5.html?sort=official) |
| ALD16A1 | SPG11 | [ARRB2](https://thebiogrid.org/229812/table/mus-musculus/arrb2.html?sort=official) | PDCD10 | [ATL3](https://thebiogrid.org/117423/table/homo-sapiens/atl3.html?sort=official) | STX2 | ALS2 | [SBSN](https://thebiogrid.org/131933/table/homo-sapiens/sbsn.html?sort=official) |
| AP5Z1 | SPG20 | [B4GALT4](https://thebiogrid.org/114245/table/homo-sapiens/b4galt4.html?sort=official) | PGAM5 | ATXN3 | [SUB1](https://thebiogrid.org/116127/table/homo-sapiens/sub1.html?sort=official) | [APOD](https://thebiogrid.org/106844/table/homo-sapiens/apod.html?sort=official) | SERPINE2 |
| [ARL6IP1](https://thebiogrid.org/116812/table/homo-sapiens/arl6ip1.html?sort=official) | SPG7 | BMP2 | [PIP](https://thebiogrid.org/111321/table/homo-sapiens/pip.html?sort=official) | ATXN3L | TEP1 | ATE1 | [SIDT2](https://thebiogrid.org/119281/table/homo-sapiens/sidt2.html?sort=official) |
| [ARL8A](https://thebiogrid.org/126084/table/homo-sapiens/arl8a.html?sort=official) | [SPRED2](https://thebiogrid.org/128344/table/homo-sapiens/spred2.html?sort=official) | BMP4 | [PKN3](https://thebiogrid.org/118978/table/homo-sapiens/pkn3.html?sort=official) | B3GALT2 | TMSB10 | [ATP2A1](https://thebiogrid.org/106977/table/homo-sapiens/atp2a1.html?sort=official) | [SLC30A9](https://thebiogrid.org/115726/table/homo-sapiens/slc30a9.html?sort=official) |
| [ATPAF2](https://thebiogrid.org/124858/table/homo-sapiens/atpaf2.html?sort=official) | [TCF12](https://thebiogrid.org/112798/table/homo-sapiens/tcf12.html?sort=official) | CALM1 | [PON2](https://thebiogrid.org/111441/table/homo-sapiens/pon2.html?sort=official) | CALR | TRIM21 | [CLDND1](https://thebiogrid.org/121164/table/homo-sapiens/cldnd1.html?sort=official) | [SOAT1](https://thebiogrid.org/112529/table/homo-sapiens/soat1.html?sort=official) |
| [CCDC102B](https://thebiogrid.org/122930/table/homo-sapiens/ccdc102b.html?sort=official) | [TCF4](https://thebiogrid.org/112787/table/homo-sapiens/tcf4.html?sort=official) | CALM2 | PPP2CA | CARS | UBC | [COX18](https://thebiogrid.org/130132/table/homo-sapiens/cox18.html?sort=official) | [SPCS1](https://thebiogrid.org/118796/table/homo-sapiens/spcs1.html?sort=official) |
| [CCDC33](https://thebiogrid.org/123126/table/homo-sapiens/ccdc33.html?sort=official) | [TFG](https://thebiogrid.org/115624/table/homo-sapiens/tfg.html?sort=official) | CCT2 | PPP2CB | CENPB | UCHL5 | [CST6](https://thebiogrid.org/107856/table/homo-sapiens/cst6.html?sort=official) | [SPCS2](https://thebiogrid.org/115133/table/homo-sapiens/spcs2.html?sort=official) |
| CD4 | TNFSF12 | CCT3 | PPP2R1A | CR1 | [UGP2](https://thebiogrid.org/113207/table/homo-sapiens/ugp2.html?sort=official) | [CTSV](https://thebiogrid.org/107895/table/homo-sapiens/ctsv.html?sort=official) | SRY |
| [CMTM5](https://thebiogrid.org/125486/table/homo-sapiens/cmtm5.html?sort=official) | [TRAF1](https://thebiogrid.org/113037/table/homo-sapiens/traf1.html?sort=official) | CCT4 | PPP2R1B | [CUL3](https://thebiogrid.org/114030/table/homo-sapiens/cul3.html?sort=official) | [UNK](https://thebiogrid.org/124536/table/homo-sapiens/unk.html?sort=official) | [DNAJC19](https://thebiogrid.org/126272/table/homo-sapiens/dnajc19.html?sort=official) | STX7 |
| [CRYAA](https://thebiogrid.org/107799/table/homo-sapiens/cryaa.html?sort=official) | [TRAF2](https://thebiogrid.org/113038/table/homo-sapiens/traf2.html?sort=official) | CCT5 | PPP2R2A | DPM2 | [UQCRQ](https://thebiogrid.org/117991/table/homo-sapiens/uqcrq.html?sort=official) | [DPY19L4](https://thebiogrid.org/130313/table/homo-sapiens/dpy19l4.html?sort=official) | SULT1C2 |
| CTDSP1 | [TRIM23](https://thebiogrid.org/106868/table/homo-sapiens/trim23.html?sort=official) | CCT6A | [PPP2R2C](https://thebiogrid.org/111514/table/homo-sapiens/ppp2r2c.html?sort=official) | [DUSP12](https://thebiogrid.org/116424/table/homo-sapiens/dusp12.html?sort=official) | USP15 | [ECEL1](https://thebiogrid.org/114820/table/homo-sapiens/ecel1.html?sort=official) | SULT1C4 |
| CTDSPL | [TRIM54](https://thebiogrid.org/121415/table/homo-sapiens/trim54.html?sort=official) | CCT7 | PPP2R4 | DYNC1H1 | USP4 | EPDR1 | THEMIS2 |
| CTPS2 | [TRIM9](https://thebiogrid.org/125280/table/homo-sapiens/trim9.html?sort=official) | CCT8 | [PRDM14](https://thebiogrid.org/122025/table/homo-sapiens/prdm14.html?sort=official) | EGLN3 | XPO7 | [FAR2](https://thebiogrid.org/120834/table/homo-sapiens/far2.html?sort=official) | [TM9SF4](https://thebiogrid.org/115121/table/homo-sapiens/tm9sf4.html?sort=official) |
| [CUTC](https://thebiogrid.org/119267/table/homo-sapiens/cutc.html?sort=official) | UBC | [COL4A5](https://thebiogrid.org/107684/table/homo-sapiens/col4a5.html?sort=official) | [PRPF31](https://thebiogrid.org/117563/table/homo-sapiens/prpf31.html?sort=official) | [ELAVL2](https://thebiogrid.org/108308/table/homo-sapiens/elavl2.html?sort=official) | ZSWIM2 | [FKBP14](https://thebiogrid.org/120362/table/homo-sapiens/fkbp14.html?sort=official) | [TNFSF9](https://thebiogrid.org/114281/table/homo-sapiens/tnfsf9.html?sort=official) |
| CYP7B1 | [XXYLT1](https://thebiogrid.org/127415/table/homo-sapiens/xxylt1.html?sort=official) | [CTNNB1](https://thebiogrid.org/107880/table/homo-sapiens/ctnnb1.html?sort=official) | [PSMD13](https://thebiogrid.org/111691/table/homo-sapiens/psmd13.html?sort=official) | EXOSC10 |  | [FKRP](https://thebiogrid.org/122565/table/homo-sapiens/fkrp.html?sort=official) | TRIM15 |
| DDHD1 | YLPM1 | CTTNBP2 | RASSF3 | FAF1 |  | [FZD2](https://thebiogrid.org/108811/table/homo-sapiens/fzd2.html?sort=official) | [TSPAN6](https://thebiogrid.org/112960/table/homo-sapiens/tspan6.html?sort=official) |
| [DHX40](https://thebiogrid.org/122790/table/homo-sapiens/dhx40.html?sort=official) | ZFYVE26 | CTTNBP2NL | [RUVBL2](https://thebiogrid.org/116067/table/homo-sapiens/ruvbl2.html?sort=official) | FAM5C |  | [FZD3](https://thebiogrid.org/113689/table/homo-sapiens/fzd3.html?sort=official) | UAP1 |
| DHX40 | [ZNF263](https://thebiogrid.org/115431/table/homo-sapiens/znf263.html?sort=official) | [DNAJA2](https://thebiogrid.org/115582/table/homo-sapiens/dnaja2.html?sort=official) | SIKE1 | GAD2 |  | [FZD6](https://thebiogrid.org/113919/table/homo-sapiens/fzd6.html?sort=official) |  |
| [DTX2](https://thebiogrid.org/125266/table/homo-sapiens/dtx2.html?sort=official) |  | [DYNLL1](https://thebiogrid.org/114206/table/homo-sapiens/dynll1.html?sort=official) | [SLC2A4](https://thebiogrid.org/112408/table/homo-sapiens/slc2a4.html?sort=official) | [GARS](https://thebiogrid.org/108887/table/homo-sapiens/gars.html?sort=official) |  | [FZD7](https://thebiogrid.org/113920/table/homo-sapiens/fzd7.html?sort=official) |  |
| DTX3L |  | [ELAVL1](https://thebiogrid.org/108309/table/homo-sapiens/elavl1.html?sort=official) | SLMAP | [GART](https://thebiogrid.org/108888/table/homo-sapiens/gart.html?sort=official) |  | [GJA1](https://thebiogrid.org/108964/table/homo-sapiens/gja1.html?sort=official) |  |
| [EFHC2](https://thebiogrid.org/123203/table/homo-sapiens/efhc2.html?sort=official) |  | [ENDOD1](https://thebiogrid.org/116689/table/homo-sapiens/endod1.html?sort=official) | [SNAI3](https://thebiogrid.org/130614/table/homo-sapiens/snai3.html?sort=official) | GEN1 |  | [GM2A](https://thebiogrid.org/109022/table/homo-sapiens/gm2a.html?sort=official) |  |
| ERLIN2 |  | [EXOC1](https://thebiogrid.org/120881/table/homo-sapiens/exoc1.html?sort=official) | [SPINK2](https://thebiogrid.org/112569/table/homo-sapiens/spink2.html?sort=official) | GFPT1 |  | GMPS |  |
| FA2H |  | FGFR1OP2 | STK24 | GLRX2 |  | [GP1BB](https://thebiogrid.org/109074/table/homo-sapiens/gp1bb.html?sort=official) |  |
| FAM114A1 |  | [FKBP15](https://thebiogrid.org/116899/table/homo-sapiens/fkbp15.html?sort=official) | STK25 | HIRIP3 |  | [GPAA1](https://thebiogrid.org/114271/table/homo-sapiens/gpaa1.html?sort=official) |  |
| [GGA2](https://thebiogrid.org/116697/table/homo-sapiens/gga2.html?sort=official) |  | [FMNL1](https://thebiogrid.org/107208/table/homo-sapiens/fmnl1.html?sort=official) | [STK26](https://thebiogrid.org/119722/table/homo-sapiens/stk26.html?sort=official) | HIST1H4A |  | [GPM6A](https://thebiogrid.org/109084/table/homo-sapiens/gpm6a.html?sort=official) |  |
| GSK3A |  | GIPC1 | STK3 | HSPA4 |  | [GRAMD1A](https://thebiogrid.org/121690/table/homo-sapiens/gramd1a.html?sort=official) |  |
| HMGCS1 |  | HEATR5A | [STK4](https://thebiogrid.org/112665/table/homo-sapiens/stk4.html?sort=official) | IGF1R |  | GRTP1 |  |
| [IKZF3](https://thebiogrid.org/116484/table/homo-sapiens/ikzf3.html?sort=official) |  | [HSPE1](https://thebiogrid.org/109568/table/homo-sapiens/hspe1.html?sort=official) | STRIP1 | INSR |  | ITGB2 |  |
| [INCA1](https://thebiogrid.org/132642/table/homo-sapiens/inca1.html?sort=official) |  | [ISCA2](https://thebiogrid.org/125808/table/homo-sapiens/isca2.html?sort=official) | STRIP2 | INSRR |  | [KIAA0196](https://thebiogrid.org/115226/table/homo-sapiens/kiaa0196.html?sort=official) |  |
| [KRT15](https://thebiogrid.org/110064/table/homo-sapiens/krt15.html?sort=official) |  | JUN | STRN | [JMJD6](https://thebiogrid.org/116817/table/homo-sapiens/jmjd6.html?sort=official) |  | [KLK7](https://thebiogrid.org/111631/table/homo-sapiens/klk7.html?sort=official) |  |
| L1CAM |  | JUNB | STRN4 | LINS |  | [LMBR1](https://thebiogrid.org/122137/table/homo-sapiens/lmbr1.html?sort=official) |  |
| MAGI2 |  | JUND | [TCF12](https://thebiogrid.org/112798/table/homo-sapiens/tcf12.html?sort=official) | [METTL18](https://thebiogrid.org/124936/table/homo-sapiens/mettl18.html?sort=official) |  | [LMF2](https://thebiogrid.org/124811/table/homo-sapiens/lmf2.html?sort=official) |  |
| MAGI3 |  | [KIAA1671](https://thebiogrid.org/124506/table/homo-sapiens/kiaa1671.html?sort=official) | TCP1 | MIS18A |  | LNX1 |  |
| MAST1 |  | [LAMA1](https://thebiogrid.org/129792/table/homo-sapiens/lama1.html?sort=official) | [TMEM126A](https://thebiogrid.org/123966/table/homo-sapiens/tmem126a.html?sort=official) | [NBR1](https://thebiogrid.org/110253/table/homo-sapiens/nbr1.html?sort=official) |  | [LPHN1](https://thebiogrid.org/116528/table/homo-sapiens/lphn1.html?sort=official) |  |
| MAST2 |  | [LIG4](https://thebiogrid.org/110169/table/homo-sapiens/lig4.html?sort=official) | [TNFRSF1A](https://thebiogrid.org/112986/table/homo-sapiens/tnfrsf1a.html?sort=official) | [NTRK1](https://thebiogrid.org/110969/table/homo-sapiens/ntrk1.html?sort=official) |  | MCM3 |  |
| MAST3 |  | [MAGEA1](https://thebiogrid.org/110274/table/homo-sapiens/magea1.html?sort=official) | [TNKS](https://thebiogrid.org/114207/table/homo-sapiens/tnks.html?sort=official) | PAGR1 |  | [MIA3](https://thebiogrid.org/131952/table/homo-sapiens/mia3.html?sort=official) |  |
| MAST4 |  | MAP2K7 | [TPTE2](https://thebiogrid.org/125031/table/homo-sapiens/tpte2.html?sort=official) | PPAT |  | MYB |  |
| [MID2](https://thebiogrid.org/116231/table/homo-sapiens/mid2.html?sort=official) |  | [MAP4K4](https://thebiogrid.org/114838/table/homo-sapiens/map4k4.html?sort=official) | TRAF3IP3 | PPM1G |  | NAMPT |  |
| MORN3 |  | MAPK10 | UBC | [PRMT3](https://thebiogrid.org/115491/table/homo-sapiens/prmt3.html?sort=official) |  | NAMPTL |  |
| [MOV10](https://thebiogrid.org/110484/table/homo-sapiens/mov10.html?sort=official) |  | MAPK8 | [XPO1](https://thebiogrid.org/113348/table/homo-sapiens/xpo1.html?sort=official) | PTPRN |  | NAPRT1 |  |
| [MTMR9](https://thebiogrid.org/122455/table/homo-sapiens/mtmr9.html?sort=official) |  | MAPK9 | [YPEL1](https://thebiogrid.org/118923/table/homo-sapiens/ypel1.html?sort=official) | PUF60 |  | [NAT14](https://thebiogrid.org/121372/table/homo-sapiens/nat14.html?sort=official) |  |
| NIPA1 |  | [MCC](https://thebiogrid.org/110333/table/homo-sapiens/mcc.html?sort=official) | [ZNF219](https://thebiogrid.org/119387/table/homo-sapiens/znf219.html?sort=official) | RAB11FIP5 |  | NIT1 |  |
| NPTN |  | [MINK1](https://thebiogrid.org/119075/table/homo-sapiens/mink1.html?sort=official) | [ZNF331](https://thebiogrid.org/120663/table/homo-sapiens/znf331.html?sort=official) | RANBP3 |  | NMNAT1 |  |
| OAT |  | MOB1A | [ZNF444](https://thebiogrid.org/120593/table/homo-sapiens/znf444.html?sort=official) | [RBM3](https://thebiogrid.org/111870/table/homo-sapiens/rbm3.html?sort=official) |  | NMNAT2 |  |
| PNPLA6 |  | MOB1B | [ZNF550](https://thebiogrid.org/127836/table/homo-sapiens/znf550.html?sort=official) | RGS18 |  | NMNAT3 |  |
| PRPS1 |  | MOB4 | ZRANB1 | [RNY1](https://thebiogrid.org/112011/table/homo-sapiens/rny1.html?sort=official) |  | PANK3 |  |
| PTEN |  | MST4 |  | [RNY3](https://thebiogrid.org/112012/table/homo-sapiens/rny3.html?sort=official) |  | PANK4 |  |
| [RABAC1](https://thebiogrid.org/115818/table/homo-sapiens/rabac1.html?sort=official) |  | [NACC1](https://thebiogrid.org/125217/table/homo-sapiens/nacc1.html?sort=official) |  | [RPL24](https://thebiogrid.org/112071/table/homo-sapiens/rpl24.html?sort=official) |  | [PIGS](https://thebiogrid.org/125082/table/homo-sapiens/pigs.html?sort=official) |  |
| [REEP6](https://thebiogrid.org/124983/table/homo-sapiens/reep6.html?sort=official) |  | [NHSL2](https://thebiogrid.org/131066/table/homo-sapiens/nhsl2.html?sort=official) |  | [RRBP1](https://thebiogrid.org/219905/table/mus-musculus/rrbp1.html?sort=official) |  | [PIGU](https://thebiogrid.org/126171/table/homo-sapiens/pigu.html?sort=official) |  |
| [RTN4](https://thebiogrid.org/121400/table/homo-sapiens/rtn4.html?sort=official) |  | [NR1H3](https://thebiogrid.org/115373/table/homo-sapiens/nr1h3.html?sort=official) |  | SEC23A |  | [POMT1](https://thebiogrid.org/115834/table/homo-sapiens/pomt1.html?sort=official) |  |
| [S100B](https://thebiogrid.org/112193/table/homo-sapiens/s100b.html?sort=official) |  | [NTRK1](https://thebiogrid.org/110969/table/homo-sapiens/ntrk1.html?sort=official) |  | SF1 |  | PUS7 |  |
| SEMA6D |  | [PARVG](https://thebiogrid.org/122059/table/homo-sapiens/parvg.html?sort=official) |  | SLC12A2 |  | RGS13 |  |
| Partner protein, color code: Black, results from BioGrid; Blue, results from STRING; Red, results from BioGrid and STRING. | | | | | | | |
