# Supplementary material for: Molecular signatures associated with tumor-specific immune response in melanoma patients treated with dendritic cell-based immunotherapy
Source: Oncotarget. 2018 Mar 30;9(24):17014–27. doi: 10.18632/oncotarget.24795 (PMC5908302; doi:10.18632/oncotarget.24795)
Supplement: Supplementary file 4 [file oncotarget-09-17014-s004.docx]

| **Supplementary Table 5. ROC curve analysis assessed by microarray time-course data.** | | | | | | | | | | | | | | | | | | | | | | |
| --- | --- | --- | --- | --- | --- | --- | --- | --- | --- | --- | --- | --- | --- | --- | --- | --- | --- | --- | --- | --- | --- | --- |
| **GEN** | **1st vaccination** | | | | | **2nd vaccination** | | | | | **3rd vaccination** | | **4th vaccination** | | | | | **DTH evaluation** | | | | |
|  | **AUC** | **Cut-off** | **S** | **E** | **p value** | **AUC** | **Cut-off** | **S** | **E** | **p value** | **AUC** | **p value** | **AUC** | **Cut-off** | **S** | **E** | **p value** | **AUC** | **Cut-off** | **S** | **E** | **p value** |
| CLEC2D | 0.46 |  |  |  | 0.88 | 0.35 |  |  |  | 0.46 | 0.80 | 0.38 | 1 | 1.23 | 100 | 100 | 0.053 | 1 | 1.17 | 100 | 100 | 0.13 |
| CXCR4 | 0.80 |  |  |  | 0.18 | 0.57 |  |  |  | 0.73 | 0.40 | 0.77 | 1 | 1.43 | 100 | 100 | 0.053 | 1 | 0.78 | 100 | 100 | 0.13 |
| FCGR2A | 0.53 |  |  |  | 0.88 | 0.65 |  |  |  | 0.46 | 0.60 | 0.77 | 1 | 1.27 | 100 | 100 | 0.053 | 0.83 |  |  |  | 0.31 |
| GIT2 | 0.53 |  |  |  | 0.88 | 0.55 |  |  |  | 0.80 | 0.80 | 0.38 | 1 | 1 | 100 | 100 | 0.053 | 1 | 0.93 | 100 | 100 | 0.13 |
| MS4A7 | 0.60 |  |  |  | 0.65 | 0.75 |  |  |  | 0.22 | 0.80 | 0.38 | 0.70 |  |  |  | 0.43 | 1 | 1.07 | 100 | 100 | 0.13 |
| PRDM1 | 0.66 |  |  |  | 0.45 | 1 | 1.215 | 100 | 100 | 0.014 | 0.80 | 0.38 | 0.80 |  |  |  | 0.24 | 1 | 1.49 | 100 | 100 | 0.13 |
| PRDX3 | 0.53 |  |  |  | 0.88 | 0.80 |  |  |  | 0.14 | 0.80 | 0.38 | 0.70 |  |  |  | 0.43 | 1 | 0.80 | 100 | 100 | 0.13 |
| SDCBP | 1 | 0.99 | 100 | 100 | 0.025 | 0.65 |  |  |  | 0.46 | 0.80 | 0.38 | 1 | 1.13 | 100 | 100 | 0.053 | 1 | 0.69 | 100 | 100 | 0.13 |
| SPG21 | 0.50 |  |  |  | 1 | 0.50 |  |  |  | 1 | 0.80 | 0.38 | 1 | 0.94 | 100 | 100 | 0.053 | 0.83 |  |  |  | 0.31 |
| VNN2 | 0.73 |  |  |  | 0.29 | 0.60 |  |  |  | 0.62 | 0.80 | 0.77 | 1 | 1.23 | 100 | 100 | 0.053 | 1 | 0.62 | 100 | 100 | 0.13 |
| Abbreviations: AUC, Area Under the Curve; S, Sensitivity; E, Specificity. | | | | | | | | | | | | | | | | | | | | | | |
